# MSIsensor-pro: Fast, Accurate, and Matched-normal-sample-free Detection of Microsatellite Instability

**DOI:** 10.1016/j.gpb.2020.02.001

**Published:** 2020-03-12

**Authors:** Peng Jia, Xiaofei Yang, Li Guo, Bowen Liu, Jiadong Lin, Hao Liang, Jianyong Sun, Chengsheng Zhang, Kai Ye

**Affiliations:** 1 School of Automation Science and Engineering, Faculty of Electronic and Information Engineering, Xi’an Jiaotong University, Xi’an 710049, China; 2 MOE Key Laboratory for Intelligent Networks & Networks Security, Faculty of Electronic and Information Engineering, Xi’an Jiaotong University, Xi’an 710049, China; 3 School of Computer Science and Technology, Faculty of Electronic and Information Engineering, Xi’an Jiaotong University, Xi’an 710049, China; 4 School of Life Science and Technology, Xi’an Jiaotong University, Xi’an 710049, China; 5 Leiden Institute of Advanced Computer Science, Leiden University, Leiden 2311 ZE, Netherlands; 6 School of Mathematics and Statistics, Xi'an Jiaotong University, Xi'an 710049, China; 7 Precision Medicine Center, the First Affiliated Hospital of Xi’an Jiaotong University, Xi’an 710061, China; 8 Jackson Laboratory for Genomic Medicine, Farmington, CT 06032, USA; 9 Genome Institute, the First Affiliated Hospital of Xi’an Jiaotong University, Xi’an 710061, China

**Keywords:** Microsatellite, Polymerase slippage, Multinomial distribution, Microsatellite instability, Tumor

## Abstract

**Microsatellite instability** (MSI) is a key biomarker for cancer therapy and prognosis. Traditional experimental assays are laborious and time-consuming, and next-generation sequencing-based computational methods do not work on leukemia samples, paraffin-embedded samples, or patient-derived xenografts/organoids, due to the requirement of matched normal samples. Herein, we developed MSIsensor-pro, an open-source single sample MSI scoring method for research and clinical applications. MSIsensor-pro introduces a **multinomial distribution** model to quantify **polymerase slippages** for each **tumor** sample and a discriminative site selection method to enable MSI detection without matched normal samples. We demonstrate that MSIsensor-pro is an ultrafast, accurate, and robust MSI calling method. Using samples with various sequencing depths and tumor purities, MSIsensor-pro significantly outperformed the current leading methods in both accuracy and computational cost. MSIsensor-pro is available at https://github.com/xjtu-omics/msisensor-pro and free for non-commercial use, while a commercial license is provided upon request.

## Introduction

Microsatellite instability (MSI) is a form of hypermutation in the microsatellites of malignancies due to a deficient DNA mismatch repair (MMR) system [1]. Significant proportions of tumor samples with MSI status are observed in colorectal cancer (CRC), stomach adenocarcinoma (STAD), and uterine corpus endometrial carcinoma (UCEC) [2], [3]. Given that MSI is an important molecular phenotype for cancers and a key biomarker for cancer immunotherapy [4], [5], [6], two gold standard detection methods, MSI-PCR and MSI-IHC, are widely used for identifying MSI clinically [7], [8]. However, both methods are laborious, time-consuming, and expensive [7], [8]. Recently, several next-generation-sequencing (NGS)-based methods have been developed, which show improved time and cost efficiency, and are highly consistent with both gold standards [2], [3], [9], [10], [11], [12], [13]. For instance, MSIsensor [10], an FDA-authorized MSI detection solution based on MSK-IMPACT [14], achieved 99.4% concordance and high sensitivity [15]. However, these NGS methods have several limitations, such as requiring matched normal samples as control (sometimes inaccessible), computational expense, and being affected by low sequencing depths and low tumor purities [7]. Particularly, due to the requirement of matched normal samples, NGS-based methods do not work on leukemia samples, paraffin embedded samples or patient-derived xenografts/organoids.

A hallmark of MSI is the enrichment of insertions or deletions in microsatellite regions initiated by polymerase slippage [16], [17] (Figure S1), which we have argued is an iterative process and described using a multinomial distribution (MND) model (Figure S2), providing promising improvements for MSI detection efficacy using NGS data. Here, we report a novel MSI calling method, MSIsensor-pro, which addresses the aforementioned limitations of current NGS-based MSI detection tools by applying an MND model to capture the intrinsic properties of polymerase slippages in a single sample. We demonstrated that MSIsensor-pro is an ultrafast, accurate, and normal sample-free MSI calling method. Moreover, it outperforms all current MSI detection methods and is robust for samples with various sequencing depths, tumor purities, and target sequencing regions.

## Method

### Data preprocessing

Whole-exome sequencing data and clinical MSI status of 1532 tumor–normal pairs were downloaded from The Cancer Genome Atlas (TCGA) [18]. The sequencing data were aligned against a human reference genome (GRCh38), and MSI was determined using the gold standards [19]. The *scan* module (default parameters) in MSIsensor [10] was used to retrieve the microsatellite regions from the human reference genome. Then, the allelic distribution of each microsatellite for each sample was extracted and used in subsequent analyses.

### Multinomial distribution model for polymerase slippage

To detect MSI without matched normal samples, we evaluated the stability of microsatellites using single samples. Based on the characteristics of allelic distribution of microsatellites in normal samples (Figures S1 and S2), we proposed that the polymerase slippage during DNA replication is an iterative process and that each step is independently accumulative. Therefore, we use multinomial distribution to model the slippage process in microsatellite sites. We use variable *x* to denote hysteresis synthesis (causing deletions; x=0), pre-synthesis (causing insertions; x=2), and normal synthesis (x=1) of each step of repeat unit synthesis, and the corresponding probabilities are denoted by *p*, *q*, and 1-p-q, respectively. Then, *x* is subjected to a multinoulli distribution, and the probability distribution function is as follows:

(1) $$pro\left(x|p,q\right)=\left\{\begin{array}{cc} p & \mathrm{if}\;x=0\\ 1-p-q & \mathrm{if}\;x=1\\ q & \mathrm{if}\;x=2 \end{array}\right)$$

Thus, for a microsatellite site with *n* repeats on the reference genome, we assume that *y* is the repeat length observed from the data. Therefore, we have:

(2) $$y=\sum_{i=1}^{n}x_{i}$$

and the probability distribution function of *y* is:

(3) $$pro\left(y|p,q\right)=\left\{\begin{array}{cc} \mathrm{pr}o_{\mathrm{ND}}+\Delta & \left(y⩽n\right)\\ \mathrm{pr}o_{\mathrm{NI}}+\Delta & \left(y>n\right) \end{array}\right)$$

where:

(4) $$\mathrm{pr}o_{\mathrm{ND}}=C_{n}^{n-y}\prod_{t=1}^{y}pro\left(x_{t}=1\right)\prod_{t=y+1}^{n}pro\left(x_{t}=0\right)$$

(5) $$\mathrm{pr}o_{\mathrm{NI}}\;\text{=}\;C_{n}^{y-n}\prod_{t=1}^{2n-y}\mathrm{pro}(x_{t}=1)\prod_{t=2n-y+1}^{n}\mathrm{pro}(x_{t}=2)$$

Here, $\mathrm{pr}o_{\mathrm{ND}}$ and $\mathrm{pr}o_{\mathrm{NI}}$ denote the probability of acquiring the observed repeat length due to deletion and insertion, respectively, with the minimum number of steps, while Δ is the probability of using more steps. Since Δ is much smaller and difficult to calculate, we ignore it in practice to preserve computational resources. For a microsatellite region spanned by *m* reads, we denote the observed repeat length as $y_{1},\;y_{2},\;\ldots y_{i}\;\ldots ,\;y_{m}$ and its distribution as $Y=\{y_{1},\;y_{2},\;\ldots y_{i}\ldots ,\;y_{m}\}$. Based on *Y*, we use the maximum likelihood estimation to compute *p* and *q* in Equation (6).

(6) $$L\left(Y|p,q\right)=\prod_{i=1}^{m}pro\left(y_{i}\right)$$

Finally, *p* and *q* can be estimated as follows:

(7) $$\left\{\begin{array}{c} p=\frac{\sum_{i=1}^{m}\left(n-y_{i}\right)}{\mathrm{nm}}\\ q=\frac{\sum_{i=n+1}^{m}\left(y_{i}-n\right)}{\mathrm{nm}} \end{array}\right)$$

The values of *p* and *q* are positively correlated with the magnitude of polymerase slippages.

### Validation of the MND model

To evaluate how well parameters *p* and *q* from the MND mimic polymerase slippages for microsatellites with various repeat lengths, we randomly selected 27,200 microsatellites from normal control samples of three cancer types in TCGA and estimated the parameters *p* and *q* for each microsatellite site. Then, the calculated *p* and *q* values (also known as the probabilities of deletion and insertion) were used to simulate allele length distribution. The sites with no significant difference (*P* < 0.05, Kolmogorov–Smirnov test) between real and simulated distribution are defined as fitted sites. Then, the percentage of fitted sites to all test sites was used to evaluate the fitness of the MND model. To investigate polymerase slippages in tumor samples, we estimated *p* and *q* for 1532 TCGA tumor samples and compared the differences between MSI and microsatellite stable (MSS) samples. In this study, only samples with status of MSI-H as determined by MSI-PCR are classified as MSI samples, whereas cancer samples with status MSS or MSI-L are classified as MSS samples, as reported previously [3]. We found that *p* discriminates between MSI and MSS samples while *q* does not, indicating that *p* is an effective metric for MSI classification.

### MSI calling of MSIsensor-pro

We used *p* (probability of deletion) from the MND model to evaluate the stability of microsatellites. To distinguish unstable sites from stable ones we determined the mean (*μ_i_*) and standard deviation (*σ_i_*) of *p* in the *i-*th microsatellite site in normal samples. Specifically, a microsatellite is classified as unstable with *p* > *μ_i_* + 3*σ_i_*. We used 1532 normal control samples from three cancer types to build the baseline. The MSI score, defined as the percentage of unstable sites within all detected sites in a sample, is used for MSI calling.

### Discriminative microsatellite site selection

To find discriminative microsatellite (DMS) sites for MSI calling, we computed the contribution of each site to MSI classification. For a given microsatellite site, the parameter *p* was used for MSI classification, and then the area under the receiver operating characteristic curve (AUC) was calculated to evaluate the contribution of this site to MSI calling. Finally, sites with AUC > 0.65 were defined as DMS sites and used for MSI calling. In this study, 340 TCGA samples were used to discover DMS sites, and all 1532 samples were used to test the performance of MSIsensor-pro.

### MSIsensor-pro performance evaluation

To assess the performance of MSIsensor-pro, we benchmarked MSIsensor-pro against MSIsensor [10], MANTIS [12], and mSINGS [11] using the 1532 TCGA tumor samples. The MSI score was used to rank sites for MSI classification, and AUC was used to evaluate the performance of each method (File S1). CPU usage, memory, and runtime for all these methods were tested on a TCGA sample, TCGA-AD-A5EJ, using a Linux machine running Ubuntu18.04 OS with Intel(R) Core (TM) i5-7500 CPU@3.40 GHz and 32-GB memory.

To compare the performances of the four methods on samples with low sequencing depths or low tumor purities, we used 178 CRC (78 MSI and 100 MSS) tumor–normal paired samples from TCGA to simulate test data. We downsampled the raw sequencing data to 5 ×, 10 ×, 20 ×, 40 ×, 60 ×, and 80 × sequencing depths and mixed different proportions of tumor and normal sequencing data to generate samples with tumor purities ranging from 5% to 80%. We called MSI for all simulated data and calculated the AUC for each method. To assess the performance of MSIsensor-pro using fewer sites, we selected microsatellite sets containing the top 1, 2, 5, 10, 20, 50, 100, 200, 500, and 1000 DMS sites for MSI calling. In addition, we randomly selected various number of microsatellites from DMS sites for MSI calling to examine the number of sites sufficient for MSI calling by MSIsensor-pro.

## Results

### Evaluation of MND model

**Figure 1:**
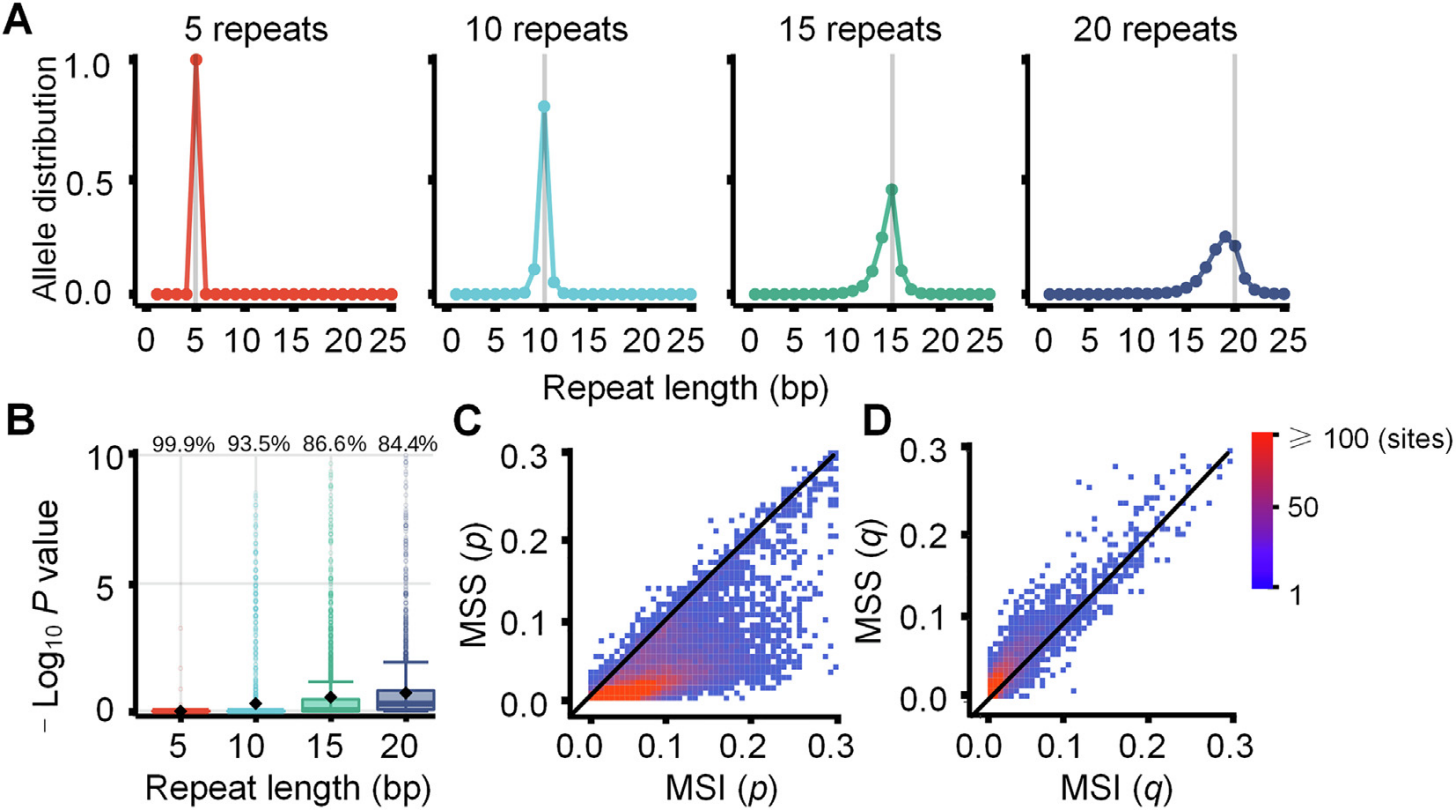
**MND model of polymerase slippages** **A.** Allele length distribution of homopolymers in normal samples. The gray vertical lines represent the repeat lengths in the human reference genome (GRCh38). **B.** The fitness of the MND model for polymerase slippages. The values on the top of boxplots represent the percentages of sites fitted (*P* < 0.05, Kolmogorov–Smirnov test) to the MND model at the respective repeat lengths. **C.** Dot plots for the means of parameter *p* (probability of deletion) in the MND model using 326 MSI and 1206 MSS samples (11,666 sites). **D.** Dot plots for the means of parameter *q* (probability of insertion) in the MND model using 326 MSI and 1206 MSS samples (11,666 sites). Dots are color-scaled according to the number of sites as shown by the color key. Dots near the diagonal lines represent sites undistinguishable between MSI and MSS. MND, multinomial distribution; MSI, microsatellite instability; MSS, microsatellite stable.

To quantitatively describe the polymerase slippages present in a single sample, we first examined the allele length distributions of 27,200 microsatellites in 1532 normal samples from TCGA [18] (Tables S1 and S2; Method). The distributions flattened (the variances became larger and the modes deviated from expectation) with increases in the repeat length of microsatellites in the reference genome (Figure 1**A**), suggesting that polymerase slippage could be an iterative process. We proposed that polymerase slippages are independently cumulative in the DNA replication process and could be modeled by the MND model. Here, we used *p* and *q* to denote the probabilities of hysteresis synthesis (causing deletions) and pre-synthesis (causing insertions), respectively, for each replication unit (Figure S2). We next estimated *p* and *q* for each microsatellite to quantify the polymerase slippage in a given allele length distribution.

To explore the characteristics of *p* and *q* in the MND model, we applied the model to 1532 TCGA normal samples. We obtained a total of 11,666 microsatellites with sufficient read coverage (>20 × ) in more than half of the samples for subsequent study (Tables S1 and S2). We found that the average probability of hysteresis synthesis, *p*, is significantly larger (*P* < 0.05, Wilcoxon rank-sum test) than that of presynthesis, *q* (Figure S3), at these sites, indicating that polymerase slippages tend to cause more deletions than insertions at microsatellites, confirming previous reports [2], [17]. To evaluate the power of our MND model for describing polymerase slippages in DNA replication, we simulated the allele length distributions at each microsatellite site with their corresponding computed *p* and *q* values, and compared them with the observed values from sequencing data. We found that the allele length distributions of the simulated data were consistent with those of observed values at 91.97% of microsatellites and the similarities between the two distributions decreased with increasing repeat length (Figures 1B and S4 and S5), confirming that the MND model is capable of describing polymerase slippages at microsatellite sites.

### Performance of MSIsensor-pro

Based on the MND model, we developed a method called MSIsensor-pro to detect MSI. We applied our MND model to 1532 TCGA tumor samples with clinical MSI status and obtained their *p* and *q* values at each microsatellite site. We found that the MSI samples have significantly larger *p* values than MSS samples (*P* < 2 × 10^16^), while *q* values in the MSI and MSS samples are not significantly different (Figures 1C, D and S6–S9). Thus, it is conceivable that either the higher incidence of polymerase slippages or failure to fix deletion errors, and therefore, the greater instability of microsatellites in MSI as opposed to MSS, could be attributed to more deletions rather than insertions [9]. Therefore, parameter *p* could evaluate the stability of each microsatellite site. MSIsensor-pro classifies the *i*-th microsatellite as unstable when its *p* is larger than *μ_i_* + 3*σ_i_*, in which *μ_i_* and *σ_i_* are the mean and standard deviation, respectively, of *p* in 1532 normal samples at the *i-th* microsatellite. The fraction of unstable sites in a given microsatellite set is used to score MSI in a tumor sample (Figure S10 and Methods).

To assess the performance of MSIsensor-pro in terms of accuracy and computational cost, we compared MSIsensor-pro against MSIsensor [10], MANTIS [12], and mSINGS [11]. Among them, MSIsensor and MANTIS require tumor–normal-paired samples, whereas mSINGS requires tumor-only samples (Tables S1 and S2; File S2). First, we applied MSIsensor-pro to 1532 TCGA tumor samples based on 11,666 preselected microsatellites to detect MSI and then compared the MSI detection accuracy with the other three methods in the same samples using AUC. We noticed that even without matched normal samples, AUC values of MSIsensor-pro are comparable to those of MSIsensor and MANTIS, but much higher than those of mSINGS (Table 1 and Table S3).

**Table 1:** **AUC obtained using four MSI detection methods for 1532 samples from TCGA**

**Method**	**Input**	**CRC (n = 588)**	**STAD (n = 412)**	**UCEC (n = 532)**	**Total (n = 1532)**
MANTIS	T–N	0.983	1.000	0.993	0.986
MSIsensor	T–N	0.981	1.000	0.988	0.989
mSINGS	T	0.594	0.711	0.634	0.594
MSIsensor-pro (all)	T	0.993	0.999	0.987	0.993
MSIsensor-pro (DMS)	T	0.997	1.000	0.990	0.994

*Note*: For MSIsensor-pro (all), all 11,666 preselected microsatellite sites were used for MSI computation; for MSIsensor-pro (DMS), only 7698 DMS sites were used for MSI computation. AUC, area under the receiver operating characteristic curve; DMS, discriminative microsatellite; CRC, colorectal cancer; STAD, stomach adenocarcinoma; UCEC, uterine corpus endometrial carcinoma; T–N, tumor–normal paired sample; T, tumor only sample.

**Figure 2:**
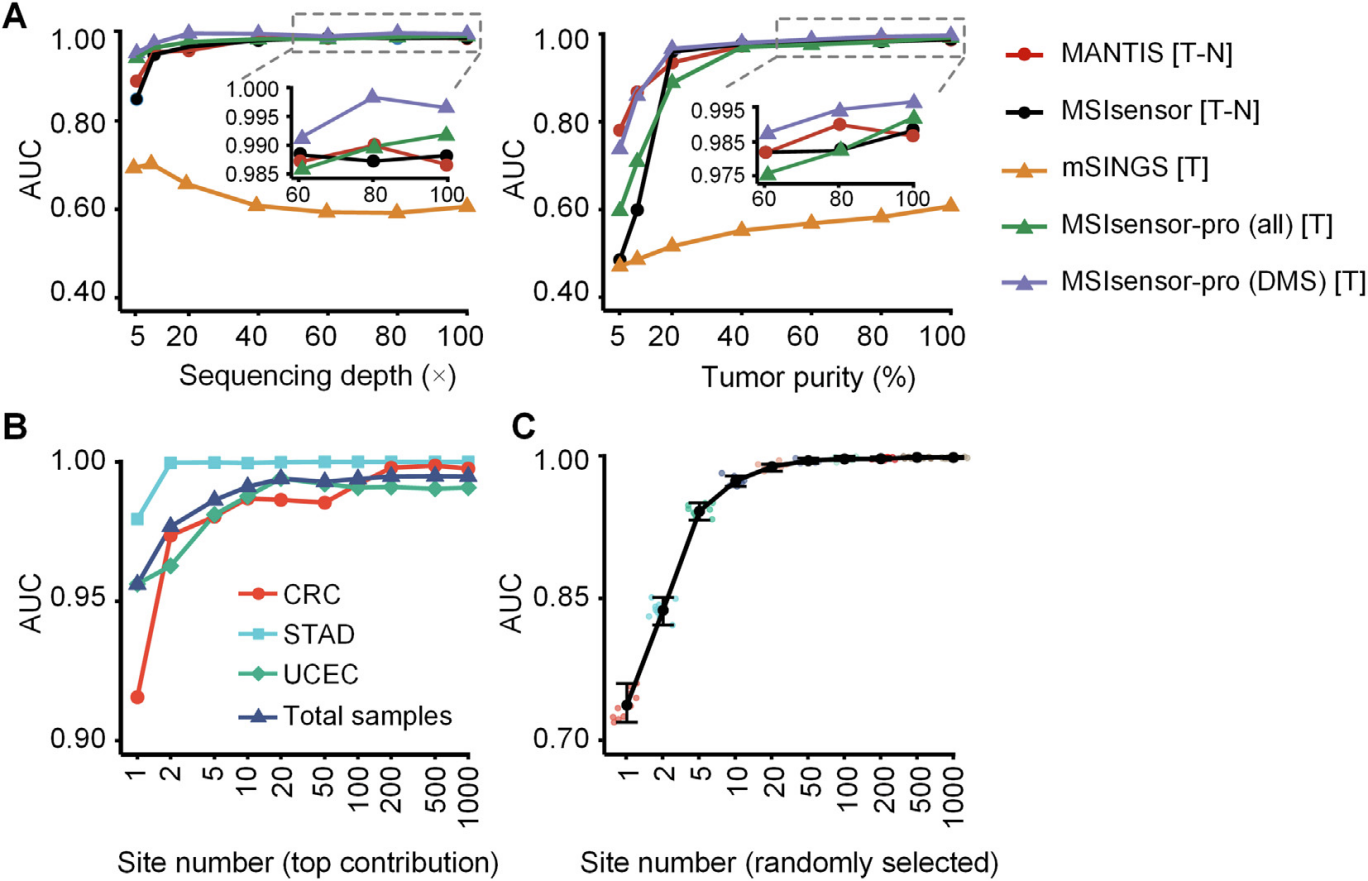
**MSI calling accuracy in TCGA dataset** **A.** AUC for four MSI detection methods across various sequencing depths (ranging from 5 × to 100 ×; left) and tumor purities (ranging from 5% to 100%; right) in 78 MSI and 100 randomly selected MSS CRC samples from TCGA. The methods tested include MSIsensor-pro, MSIsensor, MANTIS, and mSINGS. MSIsensor-pro was tested using all 11,666 preselected sites for MSIsensor-pro (all) and 7698 DMS sites for MSIsensor-pro (DMS), respectively. **B.** AUC of MSIsensor-pro using top 1–1000 contributing DMS sites for 1532 TCGA samples in total and for individual cancer types of CRC, STAD, and UCEC. AUC values approach a plateau with the top 20 contributing sites. **C.** AUC of MSIsensor-pro using 1–1000 randomly-selected DMS sites for 1532 TCGA samples. AUC values approach a plateau with 50 randomly-selected sites. These random tests were run 10 times. Data points are color-coded according to the number of DMS sites randomly selected. The black point is the mean of 10 AUC values for each group, with the top line and bottom lines of each bar representing the maximum and minimum of 10 AUCs, respectively. AUC, area under the receiver operating characteristic curve; DMS, discriminative microsatellite; CRC, colorectal cancer; STAD, stomach adenocarcinoma; UCEC, uterine corpus endometrial carcinoma. T–N, tumor–normal paired; T, tumor only.

Sequencing data from samples with low sequencing coverage or low tumor purities are common challenges for robust MSI detection in clinical applications [15]. To indicate the robustness of MSIsensor-pro for various sequencing depths or tumor purities, we evaluated the performance of all four aforementioned methods on 178 CRC samples (78 MSI and 100 MSS) in both original settings and varied sequencing depths or tumor purities. Multiple sequencing depths (5 ×, 10 ×, 20 ×, 40 ×, 60 ×, and 80 ×) resulted from simulating and downsampling the original data, while various tumor purities (5%, 10%, 20%, 40%, 60%, and 80%) were simulated by mixing the tumor and matched normal samples (Method). Across samples of diverse depths and tumor purities, AUC values of MSIsensor-pro, MSIsensor, and MANTIS were all much higher than those of mSINGS. Notably, MSIsensor-pro, requiring tumor samples only, achieved performance comparable to that of MSIsensor and MANTIS, both of which require normal–tumor-paired samples to call MSI (Figure 2**A**; Tables S4–S7). These results confirm the robustness of MSIsensor-pro and indicate that MSIsensor-pro can achieve high accuracy on samples with low sequencing depth (*e.g.*, 20 ×) or low tumor purity (*e.g.*, 40%).

To further evaluate the computational performances of all these four methods, we called MSI for a TCGA sample TCGA-AD-A5EJ (35-GB tumor and 12-GB normal bam files) using these four methods on a Linux machine running Ubuntu18.04 OS with Intel(R) Core (TM) i5-7500 CPU@3.40 GHz and 32-GB memory. MSIsensor-pro and MSIsensor required only 4 min and 15 min, respectively, thus performing significantly faster than mSINGS (94 min) and MANTIS (119 min). In addition, MSIsensor-pro consumed much less memory than MSIsensor, mSINGS, and MANTIS (Table 2; Figures S11 and S12).

**Table 2:** **Peak RAM and runtime used by four MSI detection methods for the sample TCGA-AD-A5EJ**

**Method**	**Input**	**Peak RAM (GB)**	**Runtime (min)**
MANTIS	T–N	3.712	119
MSIsensor	T–N	0.576	15
mSINGS	T	2.592	94
MSIsensor-pro (all)	T	0.032	4
MSIsensor-pro (DMS)	T	0.032	3

*Note*: Runtime is evaluated by wall clock time.

While MSIsensor-pro exhibited satisfactory all-around performance in detecting MSI using the 11,666 preselected microsatellites, these sites seemed to have an unequal contribution to MSI classifications (Figure S13). We therefore evaluated the contribution of each microsatellite based on MND parameter *p* and identified 7698 sites (Table S8) with strong contributions (AUC > 0.75), which are defined as DMS sites (Figure S13, Table S8, and Method). When only DMS sites were used, MSIsensor-pro exhibited a slight improvement compared to MSI detection using all 11,666 sites and performed superiorly to all other methods in the 1532 TCGA samples. Using DMS sites, performance of MSIsensor-pro was further enhanced with respect to sequencing data of low depths, especially for depths below 40 × (Figure 2A; Tables S4 and S5). For data of different tumor purities using DMS sites, MSIsensor-pro exhibited performance comparable to those of other tumor–normal-paired methods for tumor purities of over 40%. However, for lower tumor purities (<40%), although the performances of all methods decreased, the performance of MSIsensor-pro on DMS sites remained superior to all other methods examined (Figure 2A; Tables S6 and S7).

Since only a portion of all 11,666 sites (DMS sites) were sufficient for high performance MSI calling by MSIsensor-pro, we wonder whether an even smaller subset of DMS sites would be adequate for MSIsensor-pro to achieve similar performance, which would reduce time and cost in practical clinical applications. We therefore assessed the MSI calling performance of MSIsensor-pro on microsatellite sets from single type of tumor samples or in combination containing the top 1, 2, 5, 10, 20, 50, 100, 200, 500, and 1000 DMS sites based on their contributions. We found that even with only 1 top site, MSIsensor-pro achieved AUC values ranging 0.92–0.96 (Figure 2B; Tables S9 and S10). The performance improved with increases in the number of top sites and reached a plateau when using the top 20 sites (0.98 AUC). In addition, by testing MSIsensor-pro performance on various number of randomly selected DMS sites, we sought to identify small panels of DMS sites that are potentially effective at robust MSI calling. Indeed, we found that the AUC values for MSI detection steadily increased with growing number of randomly-selected DMS sites. When as few as 50 random sites were used, the AUC was approximately 0.98 and remained stable. Taken together, these results suggest that MSIsensor-pro could be applied to various target sequencing panels with as few as 50 sites (Figures 2C and S14; Tables S9 and S10).

## Discussion

In this study, we completely redesigned the MSI scoring strategy. By incorporating a MND model for polymerase slippage, MSIsensor-pro scores MSI on tumor samples without matched normal controls, enabling detection of MSI status on patient-derived xenografts/organoids, leukemia, and paraffin-embedded samples. In addition, MSIsensor-pro is able to score MSI using as few as 50 microsatellite sites (Figure 2C), indicating its potential to compute MSI status in cancer gene panels, stool DNA, and circulating tumor DNA from liquid biopsy samples.

MSIsensor-pro exhibits remarkable advantages in terms of both accuracy and computational cost, compared to the current leading NGS-based MSI scoring methods tested in this study, especially when processing samples with low sequencing depths or low tumor purities (Figure 2). MSIsensor-pro improves AUC values of MSI classification with tumor only samples from 0.594 (mSINGS) to 0.994 in 1532 TCGA samples (Table 1). We have also demonstrated the advantageous performance of MSIsensor-pro using data with various tumor purities (Figure 2A). We will further optimize our approach to integrate tumor purity information to our MND model for polymerase slippage.

In addition to these methodological analyses, we also examine the properties of DMS sites and find that these sites are closer to splicing sites and located in genes with higher expression than the other sites (Figures S15–S17), indicating potential roles of DMS sites in tumorigenesis.

## Code availability

MSIsensor-pro is available at https://github.com/xjtu-omics/msisensor-pro with help documentation and demo data. It is free for non-commercial use by academic, government, and non-profit/not-for-profit institutions. A commercial version of the software is available and licensed through Xi’an Jiaotong University. For more information, please contact kaiye@xjtu.edu.cn.

## Authors’ contributions

KY conceived of, designed, and supervised the study; PJ, BL, and JS developed the multinomial distribution model for polymerase slippage estimation; PJ and HL implemented the source code of MSIsensor-pro; PJ evaluated the performances of MSIsensor-pro and the other three MSI detection methods. PJ, JL, XY, LG, CZ, and KY wrote the manuscript. All authors contributed to critical revision of the manuscript, read and approved the final version.

## Competing interests

The authors declare no competing financial interests.

## Data Availability

Primary sequencing data, gold standard MSI status, and RNA expression data can be downloaded from TCGA Research Network (http://cancergenome.nih.gov/). All results generated by this study are available in Supplementary materials from the article.

## Supplementary material

Supplementary File S1 Operation parameters of four MSI detection methods.

Supplementary File S2 Operation documentation of MSIsensor-pro.

Supplementary Figure S1 Different characteristics of an MSI case between a tumor sample and the matched normal sample. This IGV screenshot of a sample with MSI-H status (TCGA-AD-A5EJ) shows that Chr3_132447304 microsatellites contain more deletions in the tumor sample than in the matched normal sample. Black line represents the deletion in the corresponding position of genome.

Supplementary Figure S2 Basic model for polymerase slippage. When the DNA strand in the top panel continues to synthesize, there are three possibilities for the next step: normal synthesis, deletion, or insertion. This process can be described as a multinoulli distribution.

Supplementary Figure S3 Boxplot of parameters p and q in the MND model. The boxplot shows that polymerase slippages accumulated with increases in the microsatellite repeat length. The values of parameter p in test microsatellites are significantly larger than those of parameter q. A rank-sum test was implemented to compare p and q at the repetitive repeat lengths. ****, *P* < 0.0001. MND, multinomial distribution.

Supplementary Figure S4 Allele length distributions of homopolymers. A. The original allele length distributions of homopolymers with 5, 10, 15, and 20 repeats in CRC, STAD, and UCEC. B. The simulated allele length distributions from calculated *p* (probability deletion) and *q* (probability insertion) in CRC, STAD, and UCEC.

Supplementary Figure S5 Performance of MND for polymerase slippage estimation in TCGA normal samples. A. *P* values for Kolmogorov-Smirnov testing between the observed allele distribution and simulated allele distribution and the values on the top of columns represent the percentages of sites fitted to the MND model at the respective repeat lengths. B. Fitness of MND for polymerase slippages, represented by the percentage of sites with *P* < 0.05 in panel (A).

Supplementary Figure S6 Parameters p and q of the MND model in different MSI status samples. The boxplot shows the mean of *p* (top panel) and *q* (bottom panel) values for each site in MSI and MSS samples in three cancer types of CRC, STAD, and UCEC. Rank-sum tests were implemented for comparison between MSI and MSS samples and the resulting *P* values were listed on tops of each cancer type.

Supplementary Figure S7 Differences in the mean of parameters p and q of the MND model between MSI and MSS samples. A. Dot plots for the means of parameter *p* (probability of deletion) in the MND model using MSI and MSS samples in 588 CRC, 412 STAD, and 532 UCEC samples of TCGA (11,666 sites). B. Dot plots for the means of parameter q (probability of insertion) in the MND model using MSI and MSS samples in 588 CRC, 412 STAD, and 532 UCEC samples of TCGA (11,666 sites). Dots are color-scaled according to the number of sites as shown by the color key. Dots near the diagonal lines represent sites undistinguishable between MSI and MSS.

Supplementary Figure S8 Density plot of parameters p and q in TCGA samples. A. Density distribution of average *p* according to 11,666 sites in MSI and MSS samples of cancer types CRC, STAD, and UCEC. B. Density distribution of average q according to 11,666 sites in CRC, STAD, and UCEC.

Supplementary Figure S9 Density plots for the standard deviations of parameters *p* and *q* in TCGA samples. A. Density distribution of standard deviations of *p* according to 11,666 sites in MSI and MSS samples of cancer types CRC, STAD, and UCEC. B. Density distribution of standard deviations *q* according to 11,666 sites in CRC, STAD, and UCEC.

Supplementary Figure S10 Workflow for MSIsensor-pro. Boxes with a red background describe basic processes for baseline building and DMS site selection. Boxes on the left and right show the flow to calculate MSIsensor-pro score of MSI for all sites and DMS sites, respectively.

Supplementary Figure S11 CPU usage along with runtime for MSI calling methods. CPU usage and runtime were tested by running TCGA-AD-A5EJ using MSIsensor-pro and three other methods, including MSIsensor, MANTIS, and mSINGS, on Ubuntu18.04 OS with an Intel(R) Core (TM) i5-7500 CPU@3.40 GHz and 32-GB memory.

Supplementary Figure S12 Memory usage along with runtime for MSI calling methods. Memory usage and runtime were tested by running TCGA-AD-A5EJ using MSIsensor-pro and three other methods, including MSIsensor, MANTIS, and mSINGS, on Ubuntu18.04 OS with an Intel(R) Core (TM) i5-7500 CPU@3.40 GHz and 32-GB memory.

Supplementary Figure S13 Density plots of site contributions for MSI calling. For each site, p of MND model is used for MSI calling, and the AUC of the site for MSI classification is used to evaluate its contribution to MSI calling.

Supplementary Figure S14 Performance of MSIsensor-pro for different site sets. Here, we randomly select 1, 2, 5, 10, 20, 50, 100, 200, 500, and 1000 DMS sites for MSI calling using MSIsensor-pro. AUC for total samples, CRC, STAD, and UCEC are calculated respectively. Data points are color-coded according to the number of DMS sites randomly selected. These random tests were run 10 times. Each data point represents the AUC of one random test for each group, and the black point is the mean of 10 AUC values, with the top line and bottom lines of each bar representing the maximum and minimum of 10 AUC values, respectively.

Supplementary Figure S15 Percentage of DMS sites in each gene region. The bar plot shows that there are more DMS sites in covered splicing sites and fewer in exonic regions.

Supplementary Figure S16 Average contribution of sites by different motif length, repeat length, and gene region. A. Homopolymers have increased contributions to MSI classification compared to sites with more than 2 repeat units. B. For homopolymers, the contributions increase with increasing repeat lengths. C. The microsatellites covering splicing sites have greater contribution to MSI classification than other regions.

Supplementary Figure S17 Characteristics of DMS sites. A. DMS sites are closer to the splicing sites than non-DMS sites. B. Genes containing DMS sites exhibit higher expression than genes covering non-DMS sites. Rank-sum tests were implemented for comparison between DMS sites and non-DMS sites. ****, *P* < 0.0001.

Supplementary Table S1 Overview of MSI status in TCGA samples.

Supplementary Table S2 Detailed information of 1532 TCGA samples.

Supplementary Table S3 MSI scores of 1532 TCGA samples using MSIsensor-pro and three other methods.

Supplementary Table S4 MSI scores of low sequencing depth data.

Supplementary Table S5 AUC for MSI calling of low sequencing depth data.

Supplementary Table S6 MSI scores of low tumor purity data.

Supplementary Table S7 AUC for MSI calling of low tumor purity data.

Supplementary Table S8 Microsatellite site information.

Supplementary Table S9 MSI scores of selected DMS sites.

Supplementary Table S10 AUC for MSI calling of selected DMS sites.
