# Supplementary material for: MSIsensor-pro: Fast, Accurate, and Matched-normal-sample-free Detection of Microsatellite Instability
Source: Genomics Proteomics Bioinformatics. 2020 Mar 12;18(1):65–71. doi: 10.1016/j.gpb.2020.02.001 (PMC7393535; doi:10.1016/j.gpb.2020.02.001)
Supplement: Supplementary File S1 — Operation parameters of four MSI detection methods. [file mmc1.docx]

**File S1 Operation parameters of four MSI detection methods**

To assess the performance of MSIsensor-pro, we applied MSIsensor-pro, MSIsensor, MANTIS, and mSINGS to TCGA samples. The following table shows the versions of each software and the parameter details.

| **Method** | **Weblink** | **Version** | **Microsatellite information** | **Minimum read coverage** | **Other parameter** |
| --- | --- | --- | --- | --- | --- |
| MSIsensor-pro | <https://github.com/xjtu-omics/msisensor-pro> | v0.1.0 | *scan* module in MSIsensor-pro | -c: 5 for sequencing coverage less than 10 × | Default |
| MSIsensor | <https://github.com/ding-lab/msisensor> | v0.1.0 | *scan* module in MSIsensor | -c: 5 for sequencing coverage less than 10 × | Default |
| MANTIS | <https://github.com/OSU-SRLab/MANTIS> | v1.0.4 | RepeatFinder | -mlc: 5 for sequencing coverage less than 10 × | Default |
| mSINGS | <https://bitbucket.org/uwlabmed/msings/src/master> | 0191.0302893 | MISA | default | Default |

*Note*: The baseline for mSINGS was built according to the instructions on its website, and the same samples were used in building the baseline.
