# Supplementary material for: MSIsensor-pro: Fast, Accurate, and Matched-normal-sample-free Detection of Microsatellite Instability
Source: Genomics Proteomics Bioinformatics. 2020 Mar 12;18(1):65–71. doi: 10.1016/j.gpb.2020.02.001 (PMC7393535; doi:10.1016/j.gpb.2020.02.001)
Supplement: Supplementary File S2 — Operation documentation of MSIsensor-pro. [file mmc2.docx]

**File S2 Operation documentation of MSIsensor-pro**

1. **Baseline**

| Baseline | | |
| --- | --- | --- |
| Function | This module builds the baseline for MSI detection with the pro module using only tumor sequencing data. To achieve this goal, sequencing data from normal samples is required (-i). | |
| Example | msisensor-pro baseline -d /path/to/reference.list -i \  /path/to/configure.txt -o /path/to/baseline/directory | |
| Parameter | Type | Explanation |
| -d | <string> | Homopolymer and microsatellite file |
| -i | <string> | Configure files for building baseline (text file) |
| -o | <string> | Output directory |
| -c | <int> | Coverage threshold for msi analysis, WXS: 20; WGS: 15, default = 20 |
| -i | <double> | A site with a detected ratio in all samples lower than this parameter will be removed in following analysis, default = 0.5 |
| -p | <int> | Minimal homopolymer size for pro analysis, default = 10 |
| -m | <int> | Maximal homopolymer size for pro analysis, default = 50 |
| -u | <int> | Span size around window for extracting reads, default = 500 |
| -s | <int> | Minimal microsatellite size for distribution analysis, default = 5 |
| -w | <int> | Maximal microsatellite size for distribution analysis, default = 40 |
| -b | <int> | Thread number for parallel computing, default = 1 |
| -x | <int> | Output homopolymer only, 0: no; 1: yes, default = 0 |
| -y | <int> | Output microsatellite only, 0: no; 1: yes, default = 0 |
| -0 | <int> | Output site with no read coverage, 1: no; 0: yes, default = 0 |
| -h | -- | Help |

1. **Pro**

| Pro | | |
| --- | --- | --- |
| Function | This module evaluates MSI using tumor only samples. Required inputs are (-d) microsatellites file and bam files (-t). | |
| Example | 1. msisensor-pro pro -d /path/to/reference.list -i 0.1 -t \   /path/to/case1_tumor_sorted.bam -o /path/to/case1_output   1. msisensor-pro pro -d /path/to/reference.list_baseline –t\   /path/to/case1_tumor_sorted.bam -o /path/to/case1_output | |
| -d | <string> | Homopolymer and microsatellites file |
| -t | <string> | Tumor bam file |
| -o | <string> | Output prefix |
| -e | <string> | Bed file, optional |
| -i | <double> | Minimal threshold for unstable site detection (for tumor only data), default = 0.1 |
| -c | <int> | Coverage threshold for msi analysis, WXS: 20; WGS: 15, default = 20 |
| -r | <string> | Choose one region, format: 1:10000000-20000000 |
| -p | <int> | Minimal homopolymer size for distribution analysis, default = 10 |
| -m | <int> | Maximal homopolymer size for distribution analysis, default = 50 |
| -s | <int> | Minimal microsatellite size for distribution analysis, default = 5 |
| -w | <int> | Maximal microsatellite size for distribution analysis, default = 40 |
| -u | <int> | Span size around window for extracting reads, default = 500 |
| -b | <int> | Thread number for parallel computing, default = 1 |
| -x | <int> | Output homopolymer only, 0: no; 1: yes, default = 0 |
| -y | <int> | Output microsatellite only, 0: no; 1: yes, default = 0 |
| -0 | <int> | Output site with no read coverage, 1: no; 0: yes, default = 0 |
| -h |  | Help |
