# Supplementary figures and images for: MSIsensor-pro: Fast, Accurate, and Matched-normal-sample-free Detection of Microsatellite Instability
Source: Genomics Proteomics Bioinformatics. 2020 Mar 12;18(1):65–71. doi: 10.1016/j.gpb.2020.02.001 (PMC7393535; doi:10.1016/j.gpb.2020.02.001)

## Slide 1
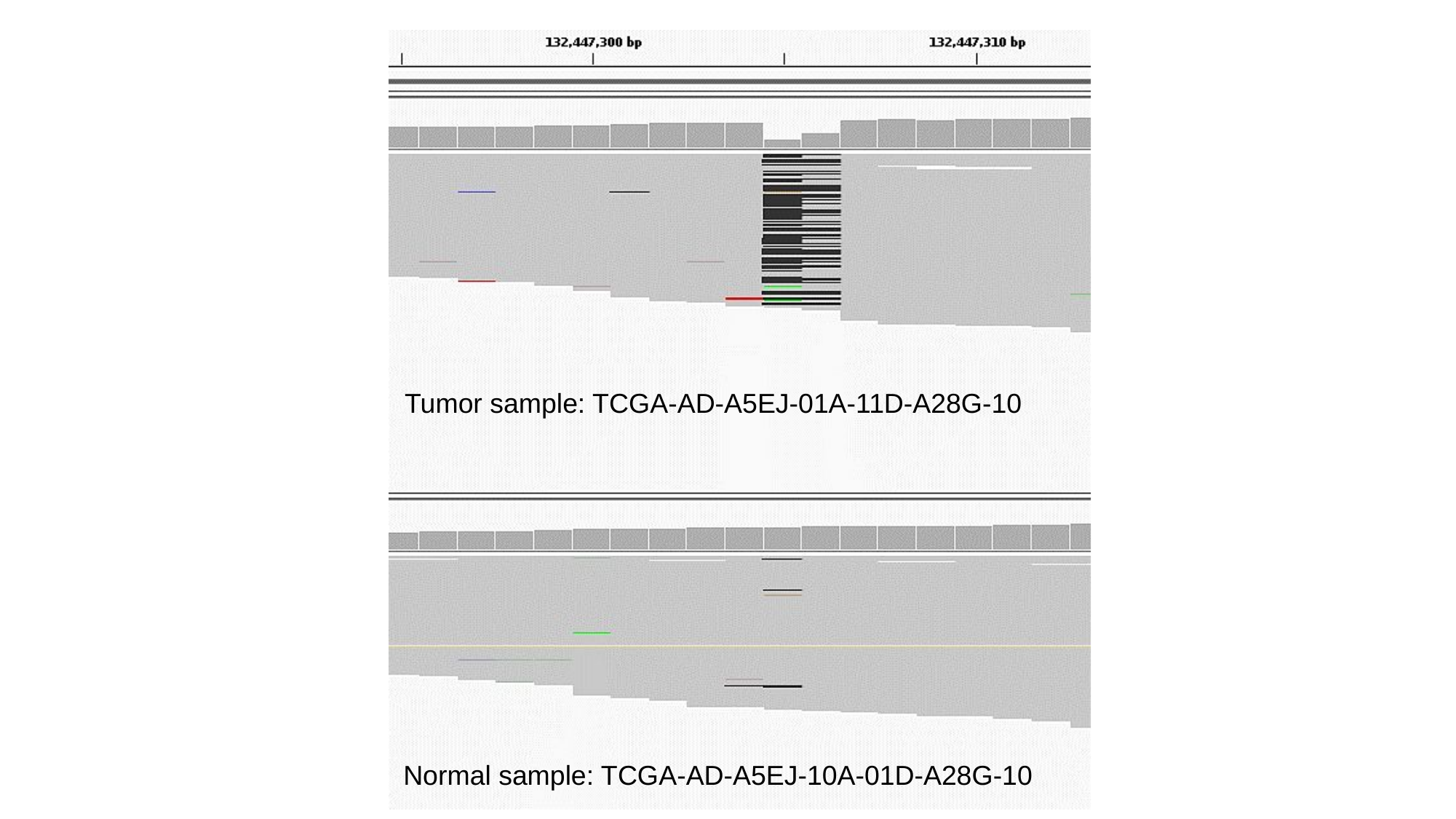

Tumor sample: TCGA-AD-A5EJ-01A-11D-A28G-10
Normal sample: TCGA-AD-A5EJ-10A-01D-A28G-10

Supplement: Supplementary Figure S1 — Different characteristics of an MSI case between a tumor sample and the matched normal sample. This IGV screenshot of a sample with MSI-H status (TCGA-AD-A5EJ) shows that Chr3_132447304 microsatellites contain more deletions in the tumor sample than in the matched normal sample. Black line represents the deletion in the corresponding position of genome. [file mmc3.ppt]

## Slide 1
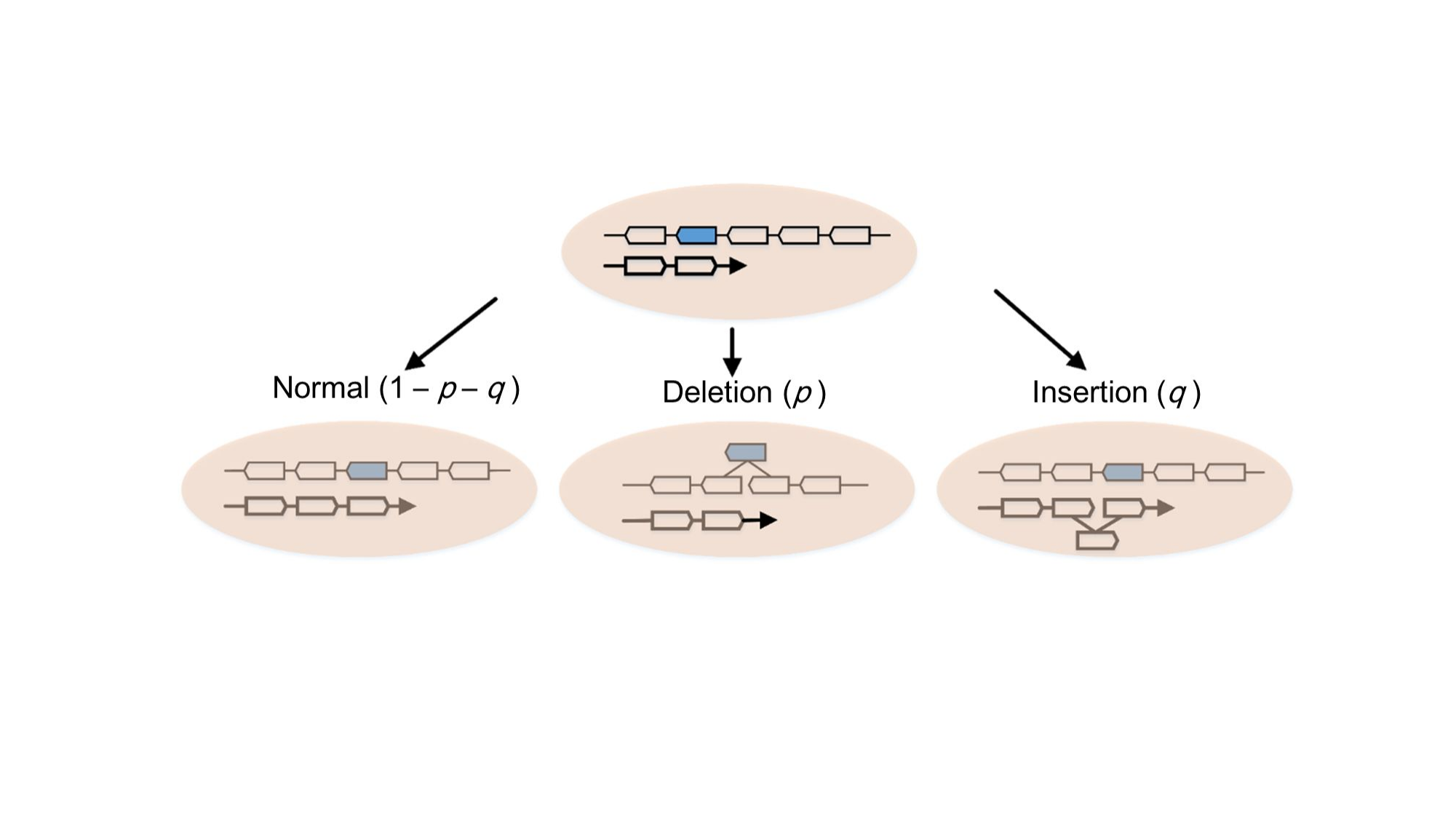

Supplement: Supplementary Figure S2 — Basic model for polymerase slippage. When the DNA strand in the top panel continues to synthesize, there are three possibilities for the next step: normal synthesis, deletion, or insertion. This process can be described as a multinoulli distribution. [file mmc4.ppt]

## Slide 1
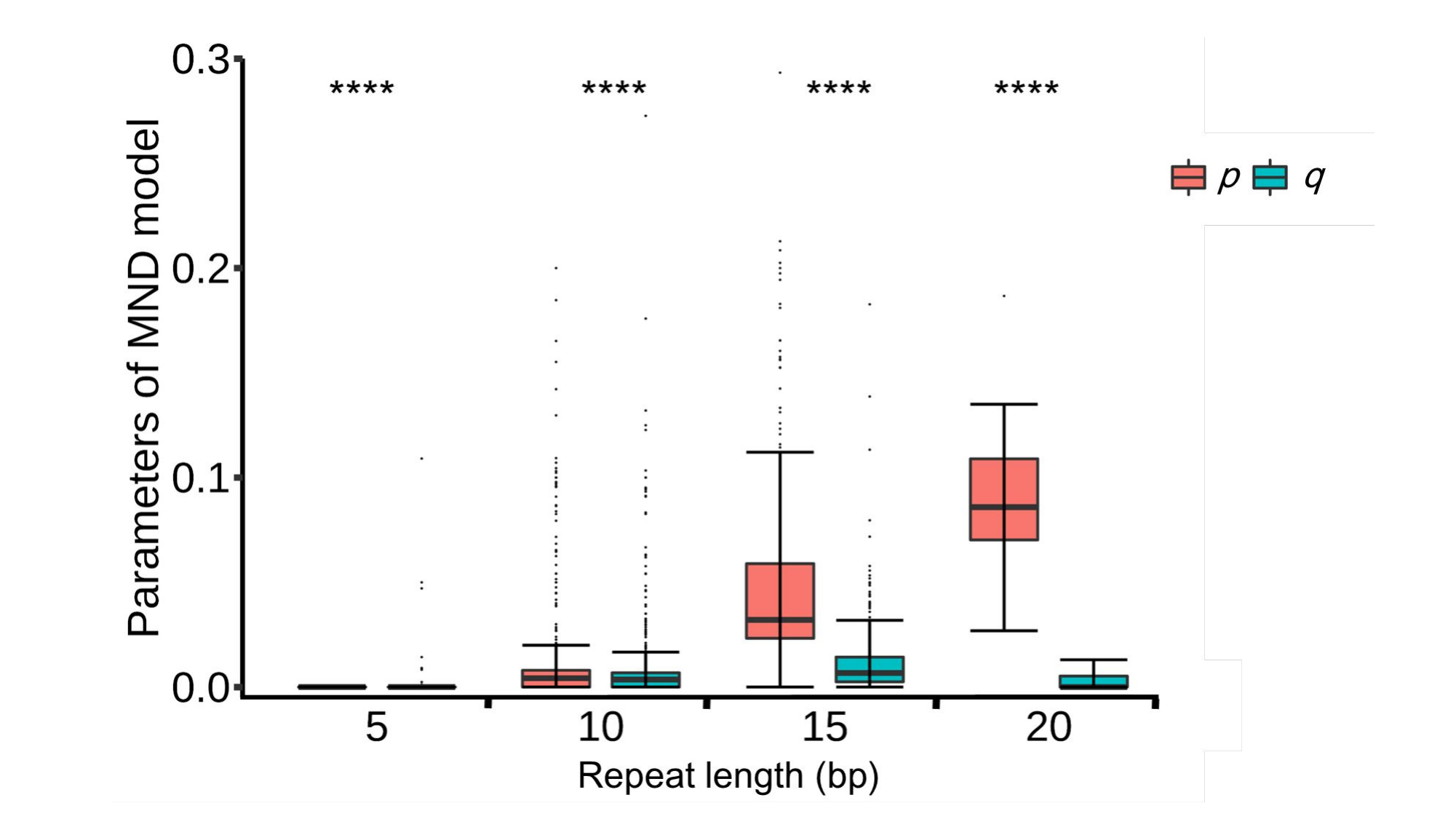

Supplement: Supplementary Figure S3 — Boxplot of parameters p and q in the MND model. The boxplot shows that polymerase slippages accumulated with increases in the microsatellite repeat length. The values of parameter p in test microsatellites are significantly larger than those of parameter q. A rank-sum test was implemented to compare p and q at the repetitive repeat lengths. ****, P < 0.0001. MND, multinomial distribution. [file mmc5.ppt]

## Slide 1
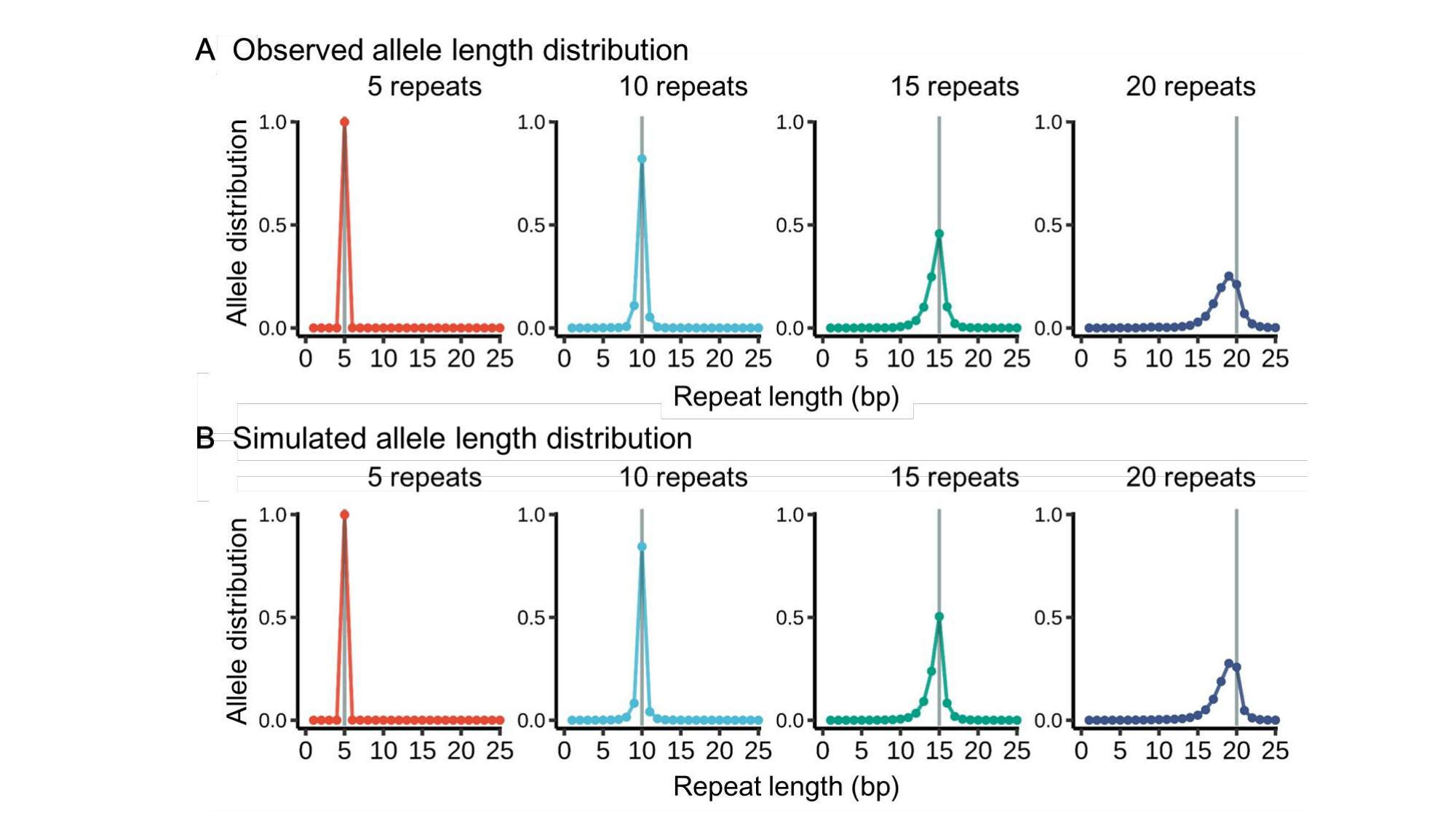

Supplement: Supplementary Figure S4 — Allele length distributions of homopolymers. A. The original allele length distributions of homopolymers with 5, 10, 15, and 20 repeats in CRC, STAD, and UCEC. B. The simulated allele length distributions from calculated p (probability deletion) and q (probability insertion) in CRC, STAD, and UCEC. [file mmc6.ppt]

## Slide 1
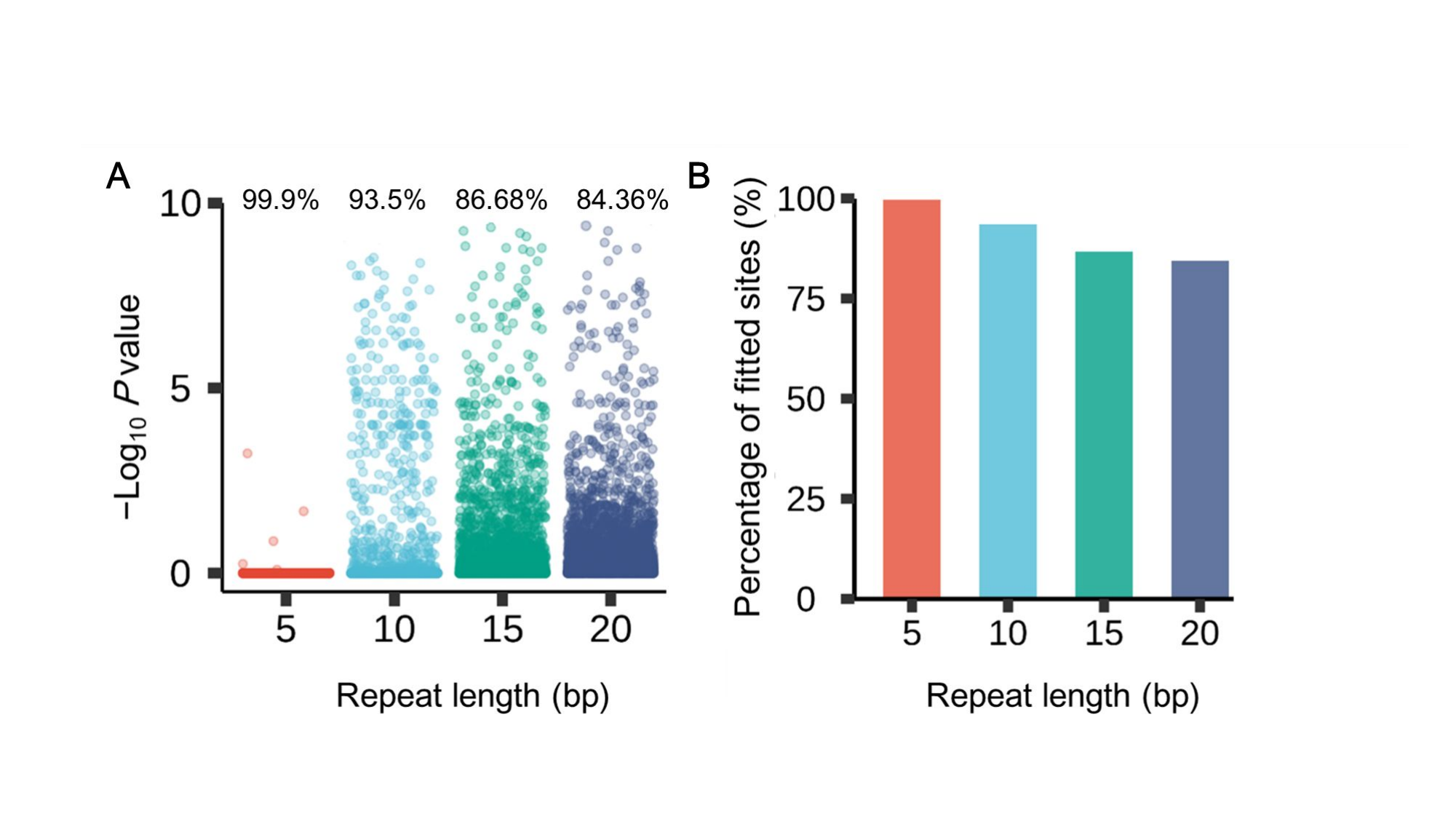

Supplement: Supplementary Figure S5 — Performance of MND for polymerase slippage estimation in TCGA normal samples. A. P values for Kolmogorov-Smirnov testing between the observed allele distribution and simulated allele distribution and the values on the top of columns represent the percentages of sites fitted to the MND model at the respective repeat lengths. B. Fitness of MND for polymerase slippages, represented by the percentage of sites with P < 0.05 in panel (A). [file mmc7.ppt]

## Slide 1
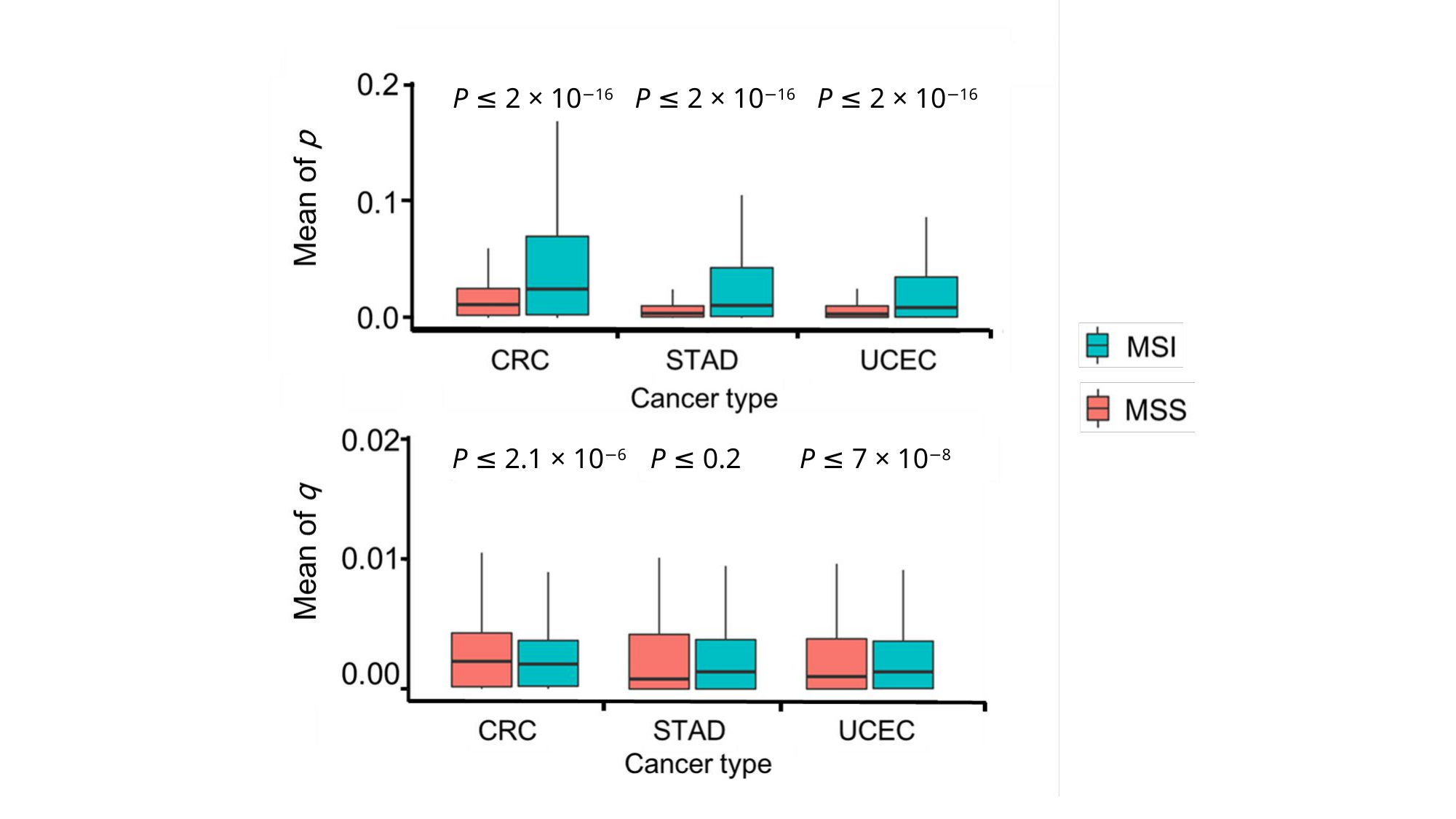

P ≤ 2 × 10−16
P ≤ 2 × 10−16
P ≤ 2 × 10−16
P ≤ 2.1 × 10−6
P ≤ 0.2
P ≤ 7 × 10−8

Supplement: Supplementary Figure S6 — Parameters p and q of the MND model in different MSI status samples. The boxplot shows the mean of p (top panel) and q (bottom panel) values for each site in MSI and MSS samples in three cancer types of CRC, STAD, and UCEC. Rank-sum tests were implemented for comparison between MSI and MSS samples and the resulting P values were listed on tops of each cancer type. [file mmc8.ppt]

## Slide 1
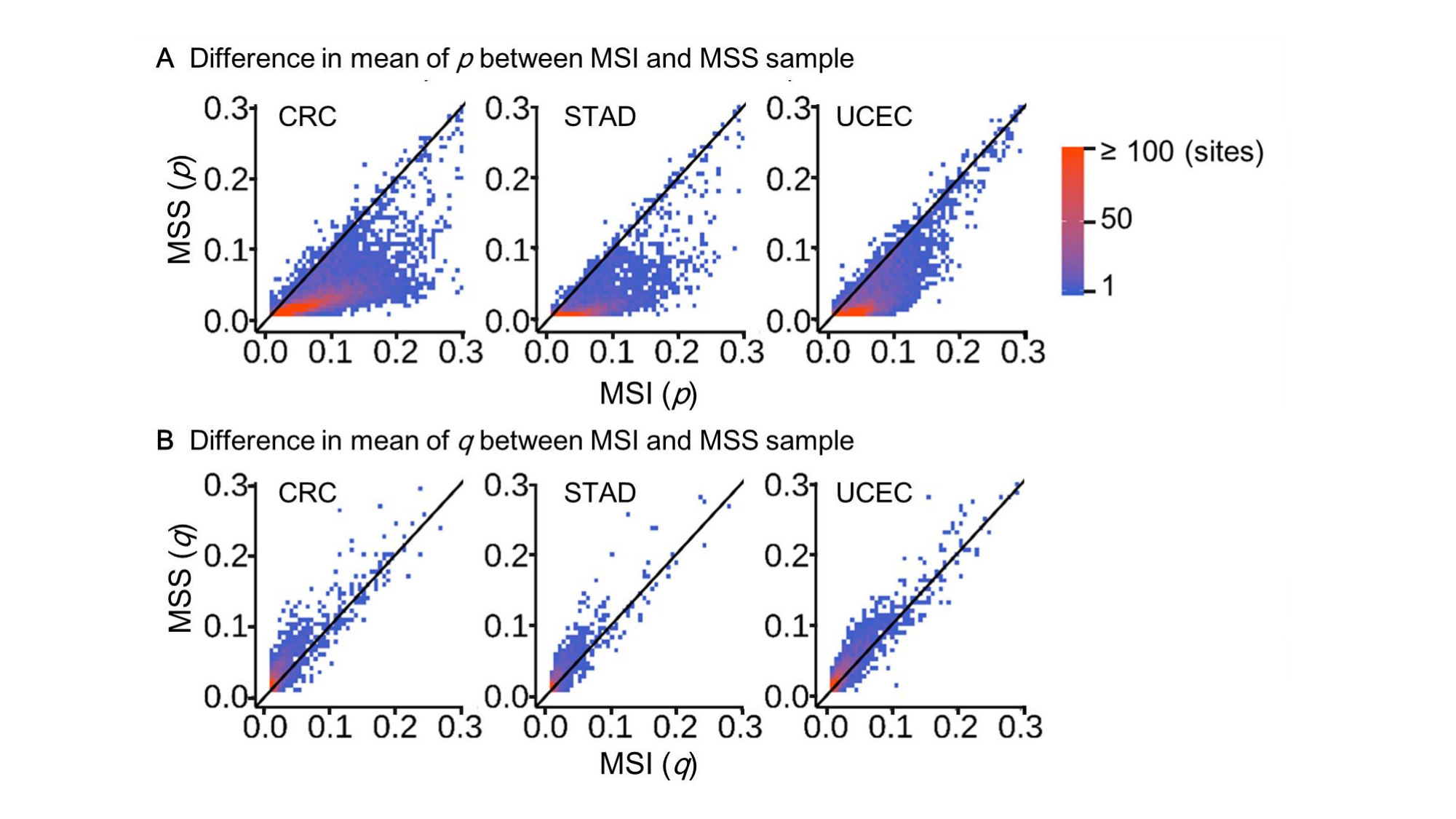

Supplement: Supplementary Figure S7 — Differences in the mean of parameters p and q of the MND model between MSI and MSS samples. A. Dot plots for the means of parameter p (probability of deletion) in the MND model using MSI and MSS samples in 588 CRC, 412 STAD, and 532 UCEC samples of TCGA (11,666 sites). B. Dot plots for the means of parameter q (probability of insertion) in the MND model using MSI and MSS samples in 588 CRC, 412 STAD, and 532 UCEC samples of TCGA (11,666 sites). Dots are color-scaled according to the number of sites as shown by the color key. Dots near the diagonal lines represent sites undistinguishable between MSI and MSS. [file mmc9.ppt]

## Slide 1
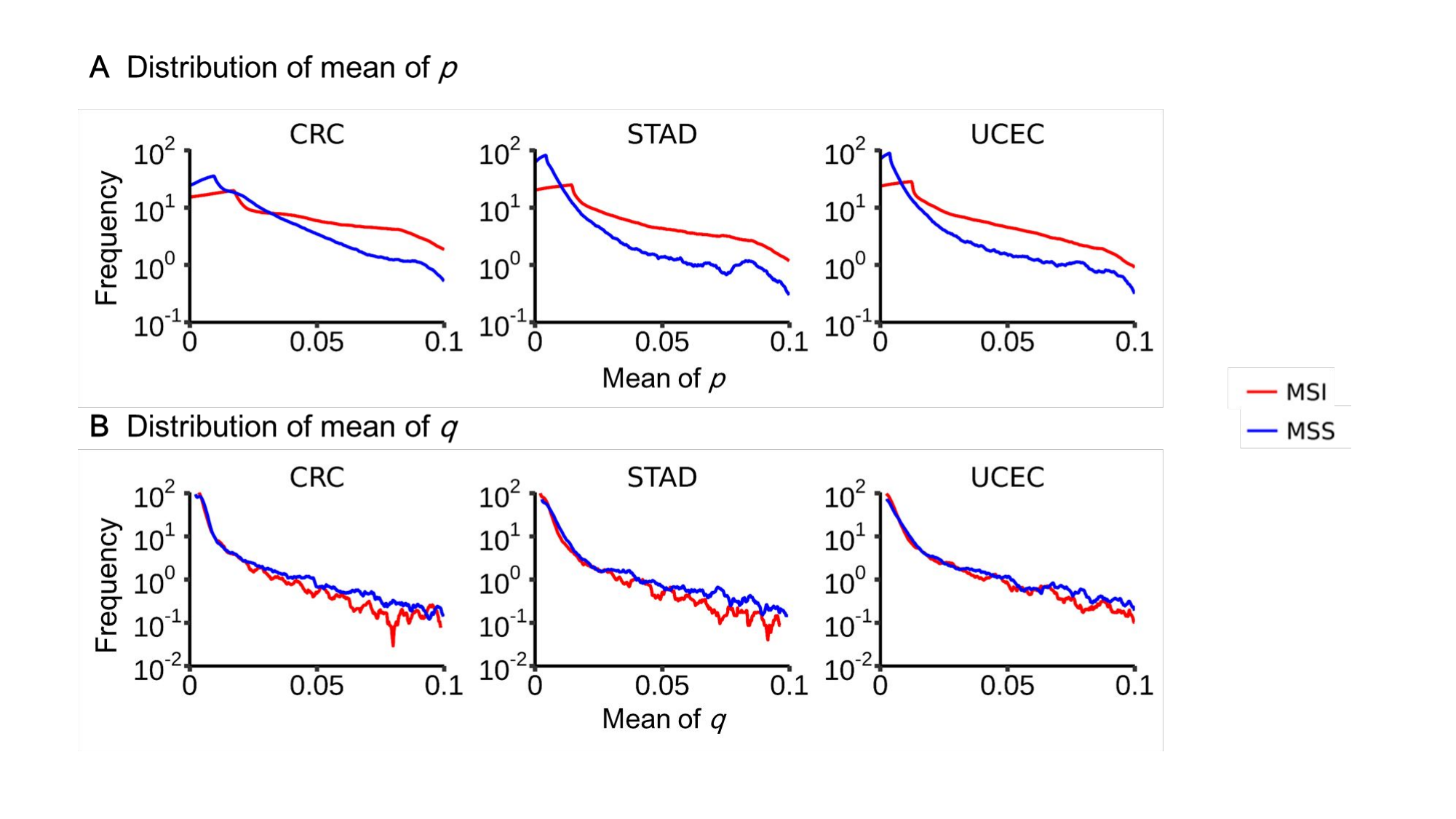

Supplement: Supplementary Figure S8 — Density plot of parameters p and q in TCGA samples. A. Density distribution of average p according to 11,666 sites in MSI and MSS samples of cancer types CRC, STAD, and UCEC. B. Density distribution of average q according to 11,666 sites in CRC, STAD, and UCEC. [file mmc10.ppt]

## Slide 1
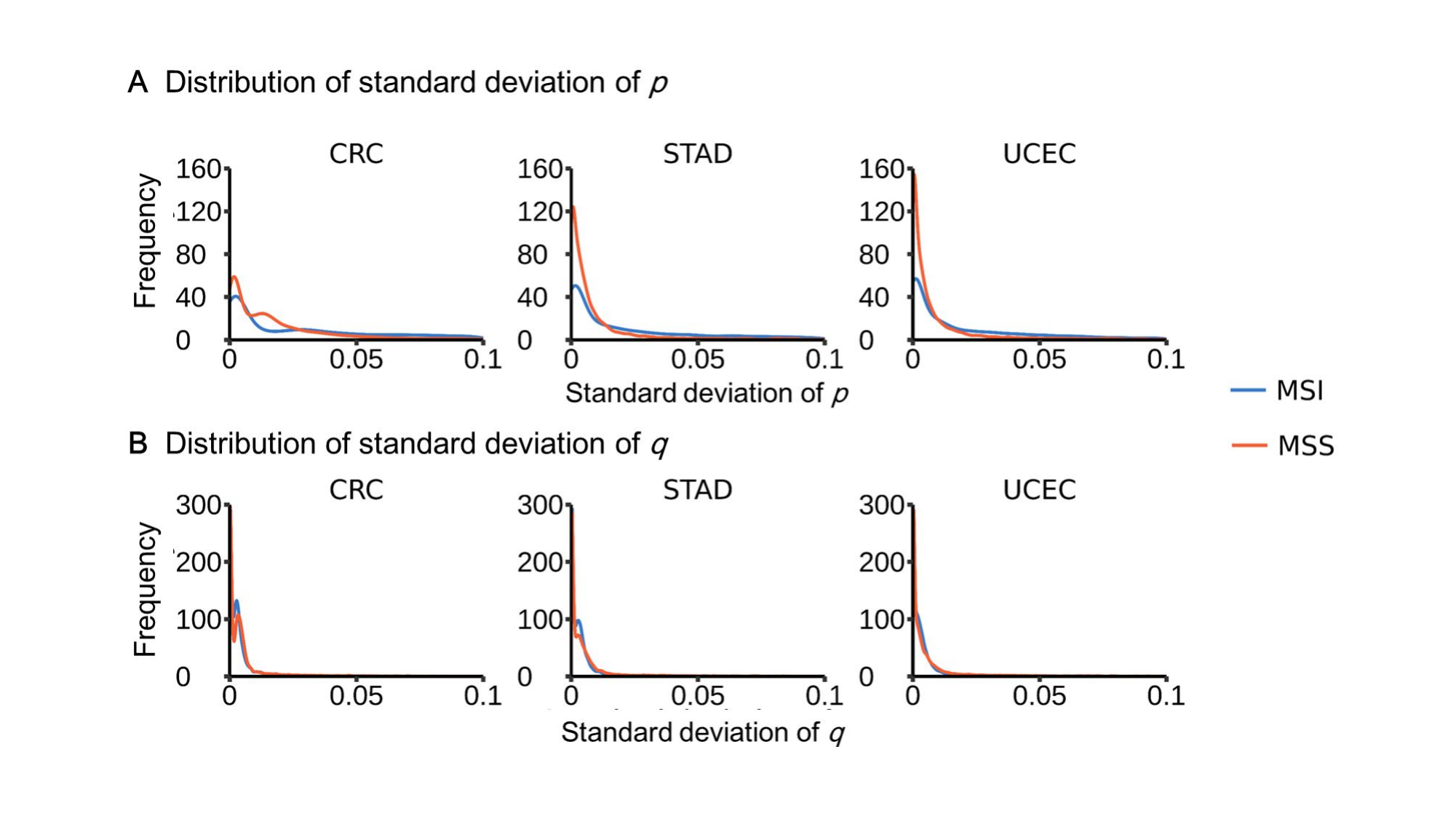

Supplement: Supplementary Figure S9 — Density plots for the standard deviations of parameters p and q in TCGA samples. A. Density distribution of standard deviations of p according to 11,666 sites in MSI and MSS samples of cancer types CRC, STAD, and UCEC. B. Density distribution of standard deviations q according to 11,666 sites in CRC, STAD, and UCEC. [file mmc11.ppt]

## Slide 1
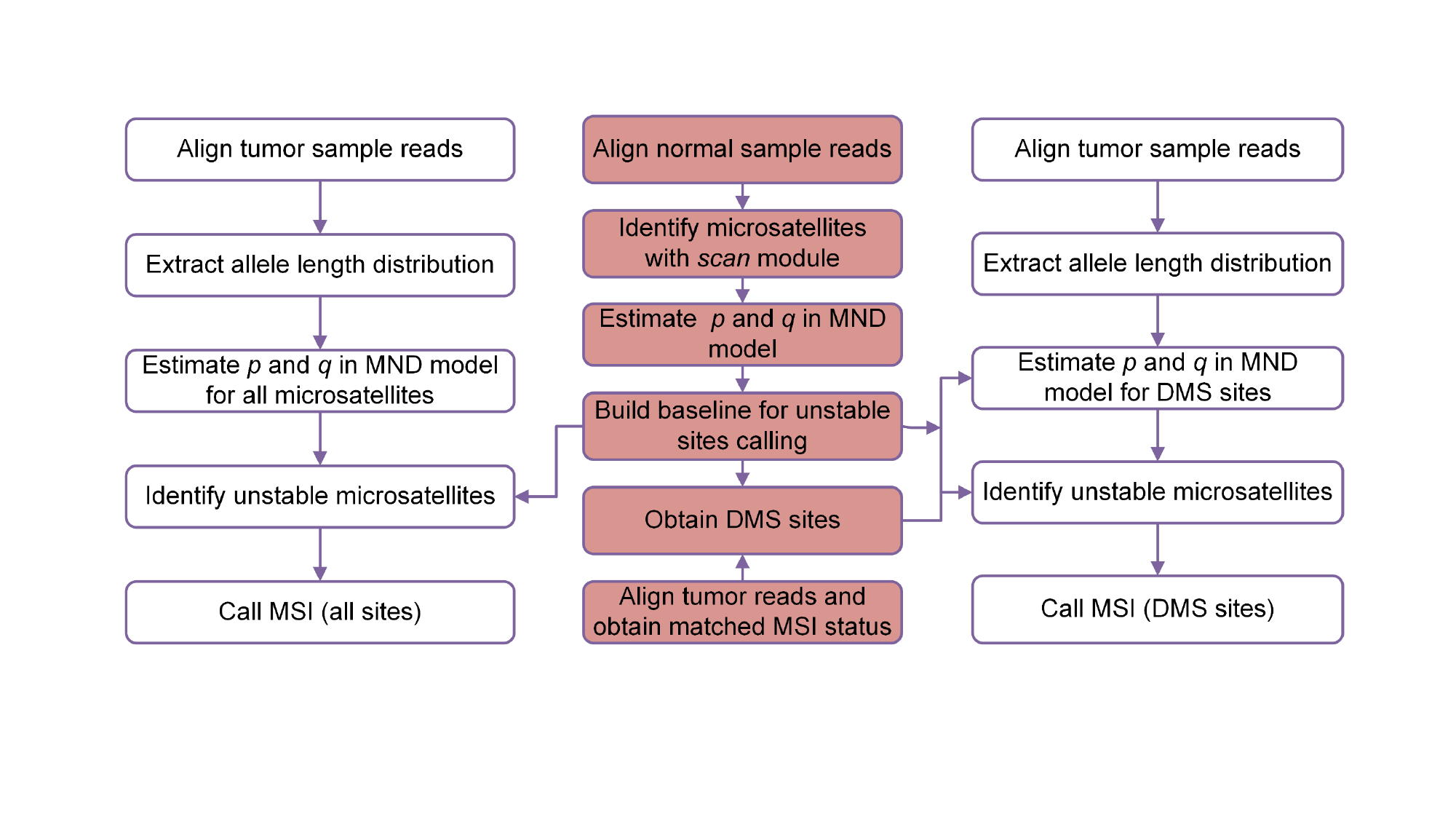

Supplement: Supplementary Figure S10 — Workflow for MSIsensor-pro. Boxes with a red background describe basic processes for baseline building and DMS site selection. Boxes on the left and right show the flow to calculate MSIsensor-pro score of MSI for all sites and DMS sites, respectively. [file mmc12.ppt]

## Slide 1
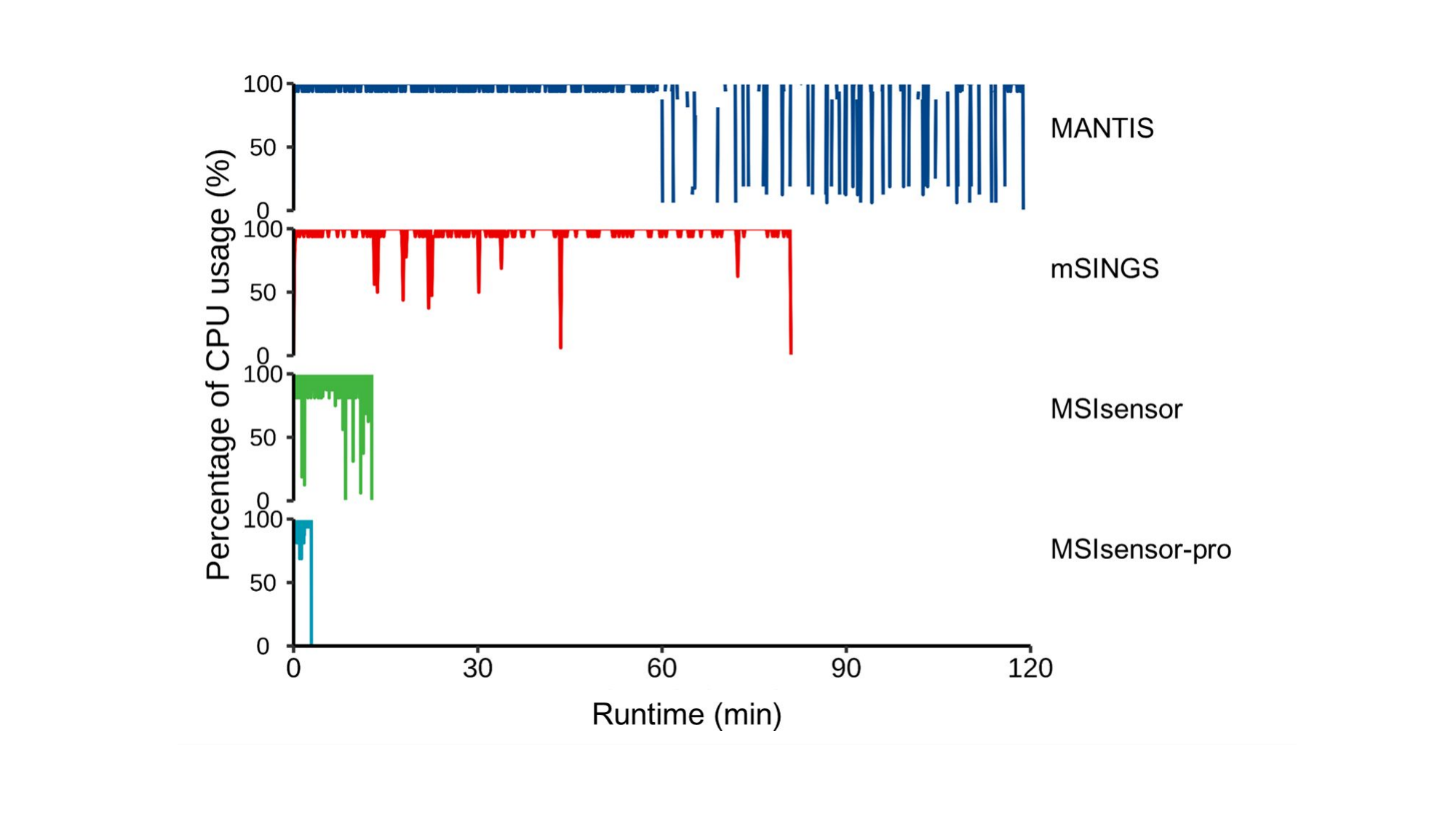

Supplement: Supplementary Figure S11 — CPU usage along with runtime for MSI calling methods. CPU usage and runtime were tested by running TCGA-AD-A5EJ using MSIsensor-pro and three other methods, including MSIsensor, MANTIS, and mSINGS, on Ubuntu18.04 OS with an Intel(R) Core (TM) i5-7500 CPU@3.40 GHz and 32-GB memory. [file mmc13.ppt]

## Slide 1
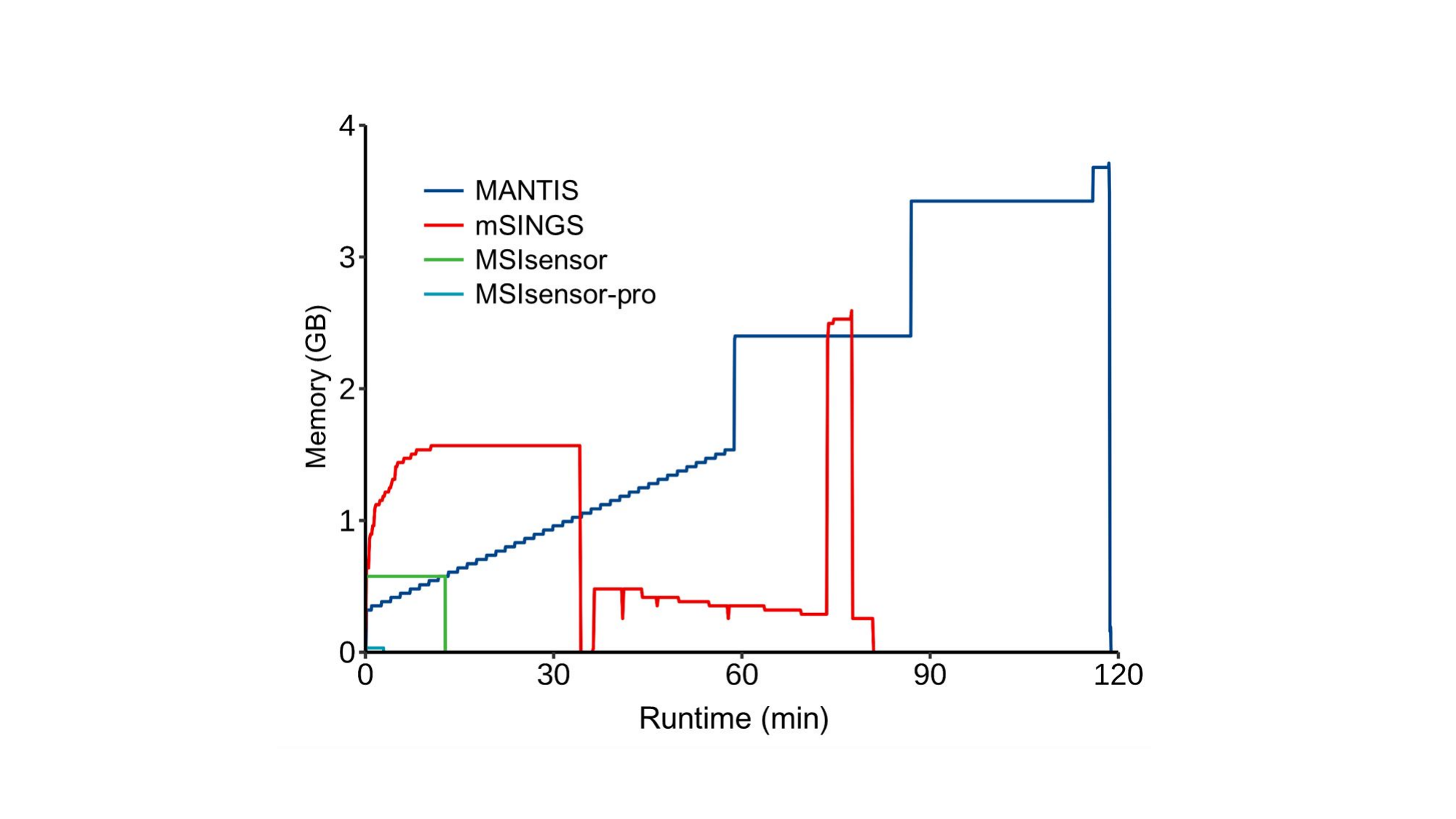

Supplement: Supplementary Figure S12 — Memory usage along with runtime for MSI calling methods. Memory usage and runtime were tested by running TCGA-AD-A5EJ using MSIsensor-pro and three other methods, including MSIsensor, MANTIS, and mSINGS, on Ubuntu18.04 OS with an Intel(R) Core (TM) i5-7500 CPU@3.40 GHz and 32-GB memory. [file mmc14.ppt]

## Slide 1
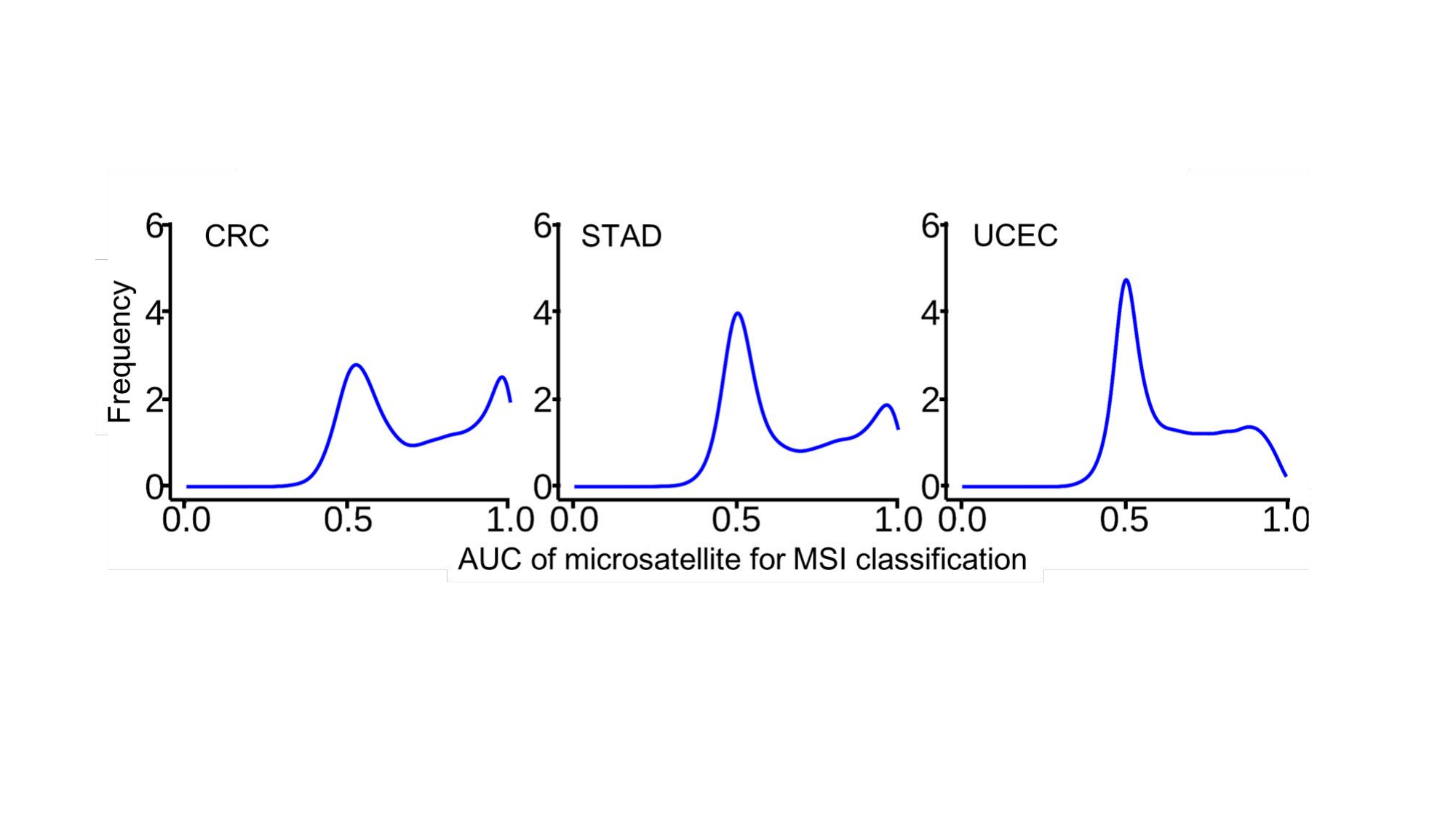

Supplement: Supplementary Figure S13 — Density plots of site contributions for MSI calling. For each site, p of MND model is used for MSI calling, and the AUC of the site for MSI classification is used to evaluate its contribution to MSI calling. [file mmc15.ppt]

## Slide 1
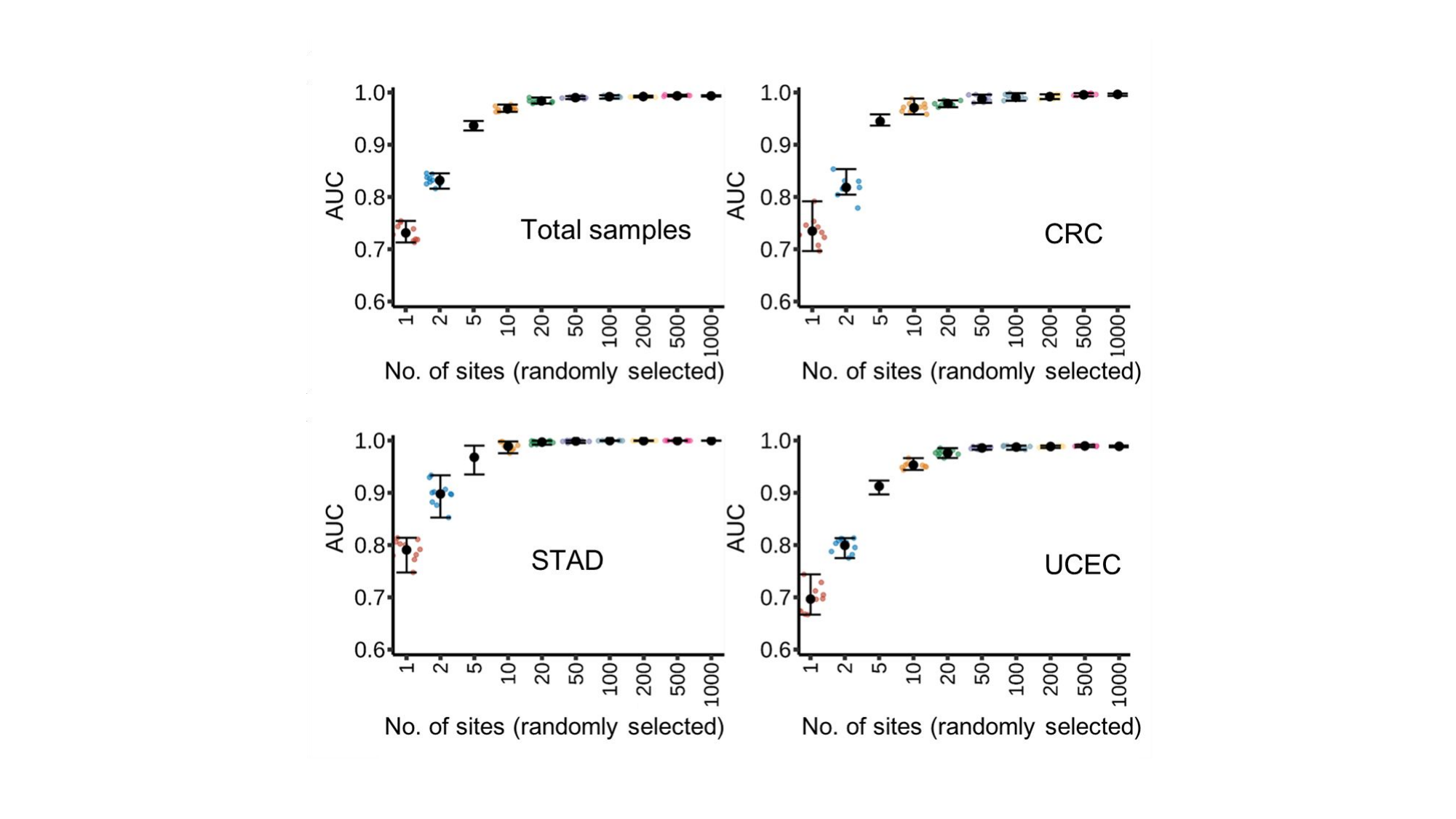

Supplement: Supplementary Figure S14 — Performance of MSIsensor-pro for different site sets. Here, we randomly select 1, 2, 5, 10, 20, 50, 100, 200, 500, and 1000 DMS sites for MSI calling using MSIsensor-pro. AUC for total samples, CRC, STAD, and UCEC are calculated respectively. Data points are color-coded according to the number of DMS sites randomly selected. These random tests were run 10 times. Each data point represents the AUC of one random test for each group, and the black point is the mean of 10 AUC values, with the top line and bottom lines of each bar representing the maximum and minimum of 10 AUC values, respectively. [file mmc16.ppt]

## Slide 1
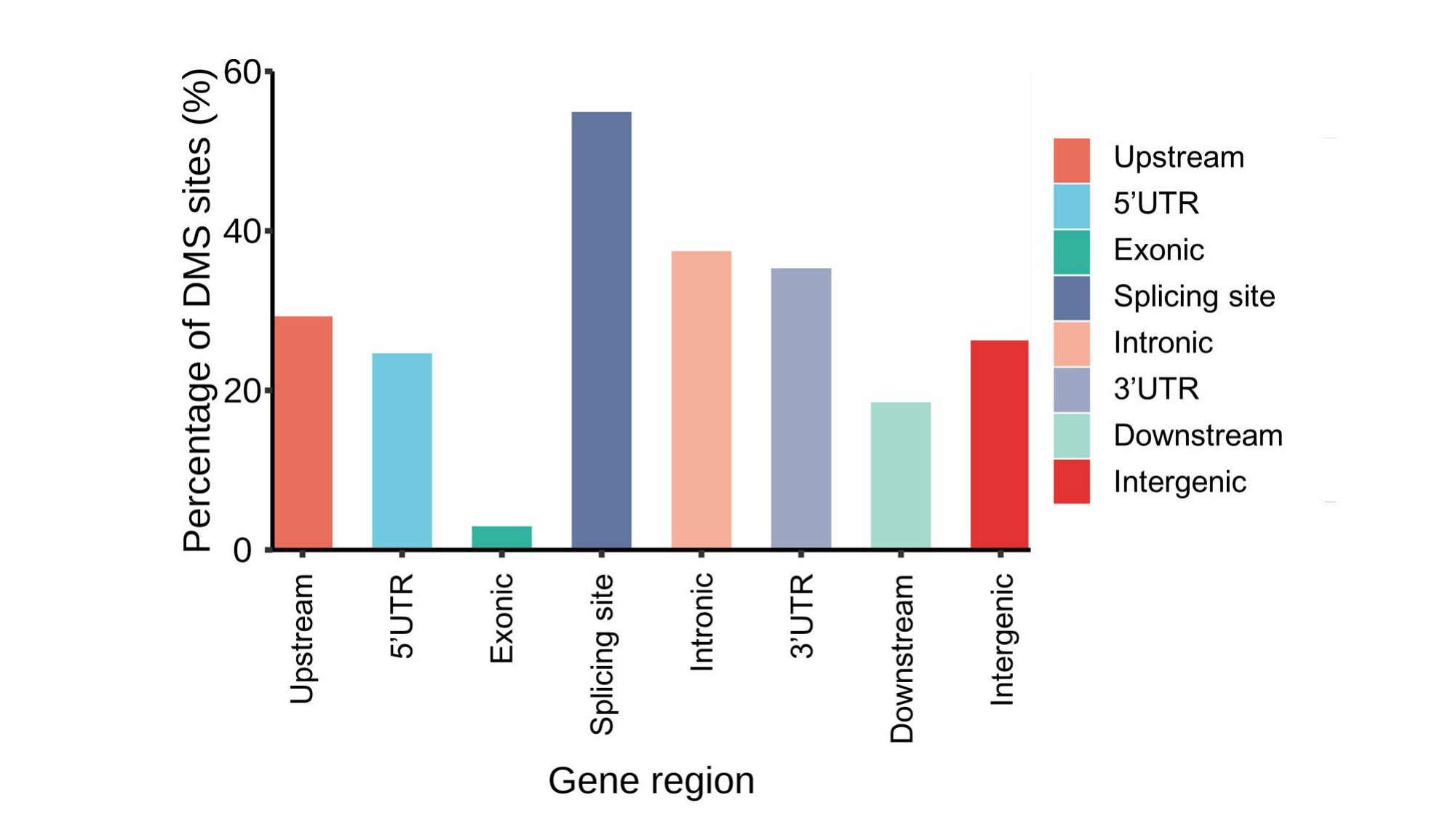

Supplement: Supplementary Figure S15 — Percentage of DMS sites in each gene region. The bar plot shows that there are more DMS sites in covered splicing sites and fewer in exonic regions. [file mmc17.ppt]

## Slide 1
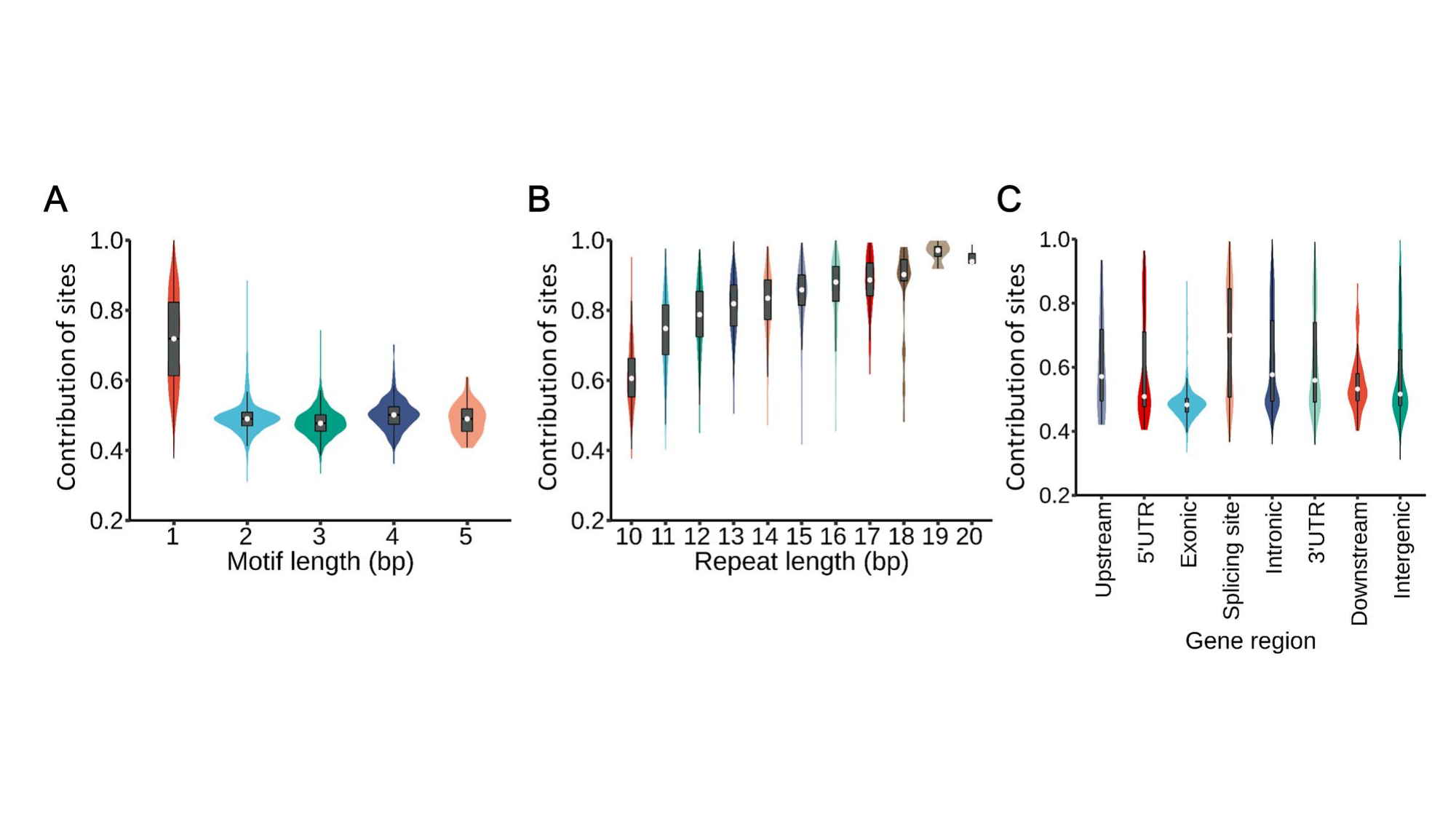

Supplement: Supplementary Figure S16 — Average contribution of sites by different motif length, repeat length, and gene region. A. Homopolymers have increased contributions to MSI classification compared to sites with more than 2 repeat units. B. For homopolymers, the contributions increase with increasing repeat lengths. C. The microsatellites covering splicing sites have greater contribution to MSI classification than other regions. [file mmc18.ppt]

## Slide 1
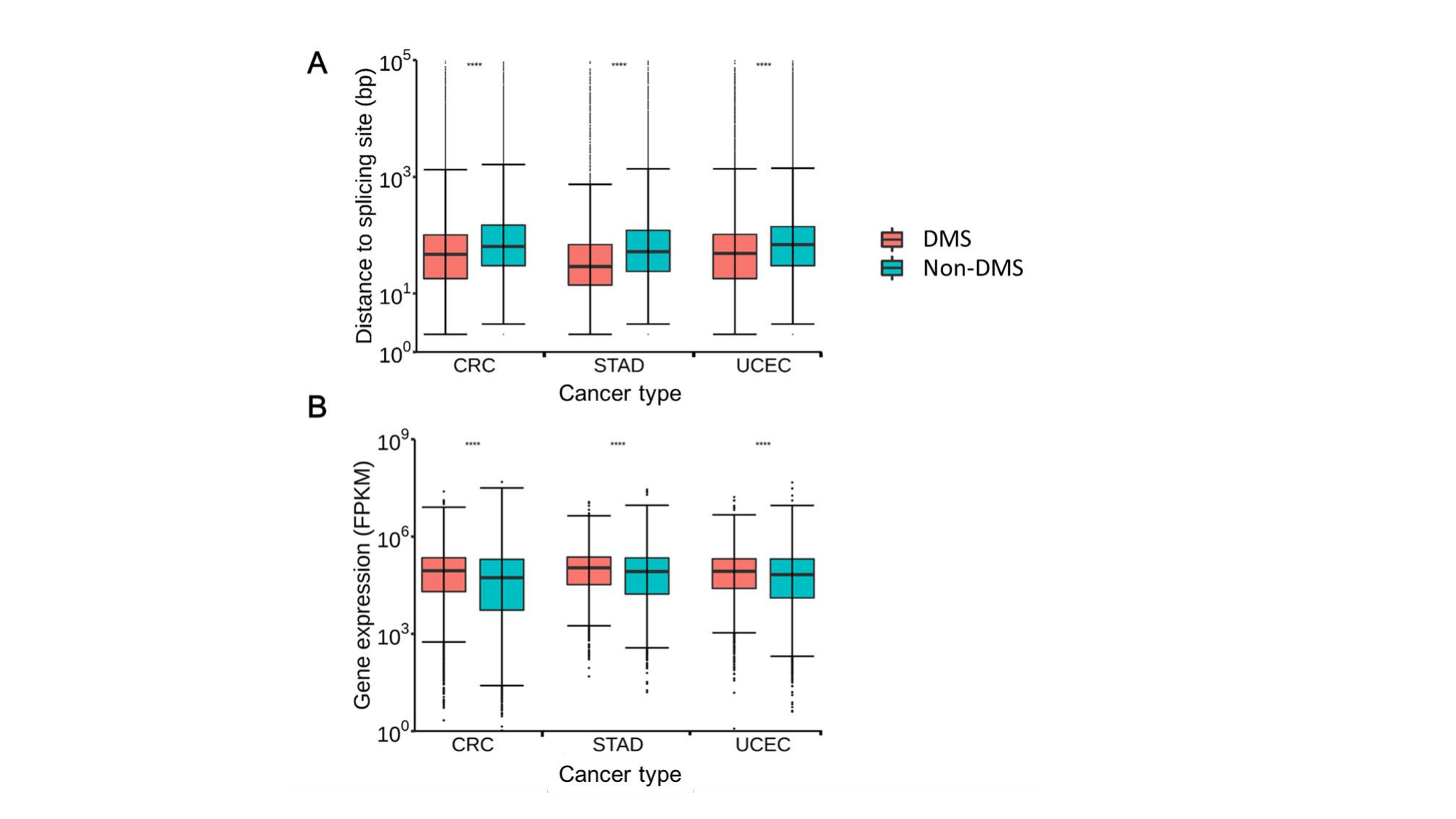

Supplement: Supplementary Figure S17 — Characteristics of DMS sites. A. DMS sites are closer to the splicing sites than non-DMS sites. B. Genes containing DMS sites exhibit higher expression than genes covering non-DMS sites. Rank-sum tests were implemented for comparison between DMS sites and non-DMS sites. ****, P < 0.0001. [file mmc19.ppt]
