# Supplementary material for: MSIsensor-pro: Fast, Accurate, and Matched-normal-sample-free Detection of Microsatellite Instability
Source: Genomics Proteomics Bioinformatics. 2020 Mar 12;18(1):65–71. doi: 10.1016/j.gpb.2020.02.001 (PMC7393535; doi:10.1016/j.gpb.2020.02.001)
Supplement: Supplementary Table S1 — Overview of MSI status in TCGA samples. [file mmc20.docx]

**Table S1 Overview of MSI status in TCGA samples**

| **Cancer type** | **MSI-H** | **MSS and MSI-L** | **Total** |
| --- | --- | --- | --- |
| CRC | 28/78 | 109/510 | 137/588 |
| STAD | 28/80 | 69/332 | 97/412 |
| UCEC | 59/168 | 47/364 | 106/532 |
| Total | 115/326 | 225/1206 | 340/1532 |

*Note*: Sample numbers are presented as N/M for each group; M represents the total number of samples at the indicated MSI status and N is the number of samples for the importance determination of microsatellites. MSI status (MSI-H, MSI-L, and MSS) was determined by PCR. In this study, cancer samples with status MSI-H are classified as MSI samples, whereas cancer samples with status MSS or MSI-L are classified as MSS samples. MSI-H, microsatellite instability high; MSI-L, microsatellite instability low; MSS, microsatellite stable.
