# Supplementary material for: MSIsensor-pro: Fast, Accurate, and Matched-normal-sample-free Detection of Microsatellite Instability
Source: Genomics Proteomics Bioinformatics. 2020 Mar 12;18(1):65–71. doi: 10.1016/j.gpb.2020.02.001 (PMC7393535; doi:10.1016/j.gpb.2020.02.001)
Supplement: Supplementary Table S5 — AUC for MSI calling of low sequencing depth data. [file mmc24.docx]

**Table S5 AUC for MSI calling of low sequencing depth data**

| **Sequencing depth (×)** | **mSINGS** | **MANTIS** | **MSIsensor** | **MSIsensor-pro (all)** | **MSIsensor-pro (DMS)** |
| --- | --- | --- | --- | --- | --- |
| 5 | 0.6932 | 0.8902 | 0.8491 | 0.9425 | 0.9535 |
| 10 | 0.7022 | 0.9553 | 0.9500 | 0.9657 | 0.9753 |
| 20 | 0.6575 | 0.9592 | 0.9678 | 0.9786 | 0.9977 |
| 40 | 0.6085 | 0.9868 | 0.9824 | 0.9849 | 0.9971 |
| 60 | 0.5938 | 0.9871 | 0.9883 | 0.9856 | 0.9912 |
| 80 | 0.5923 | 0.9899 | 0.9872 | 0.9897 | 0.9983 |
| 100 | 0.6062 | 0.9866 | 0.9882 | 0.9919 | 0.9965 |
