# Supplementary material for: MSIsensor-pro: Fast, Accurate, and Matched-normal-sample-free Detection of Microsatellite Instability
Source: Genomics Proteomics Bioinformatics. 2020 Mar 12;18(1):65–71. doi: 10.1016/j.gpb.2020.02.001 (PMC7393535; doi:10.1016/j.gpb.2020.02.001)
Supplement: Supplementary Table S7 — AUC for MSI calling of low tumor purity data. [file mmc26.docx]

**Table S7 AUC for MSI calling of low tumor purity data**

| **Tumor purity (%)** | **mSINGS** | **MANTIS** | **MSIsensor** | **MSIsensor-pro (all)** | **MSIsensor-pro (DMS)** |
| --- | --- | --- | --- | --- | --- |
| 5 | 0.4718 | 0.7807 | 0.4853 | 0.5971 | 0.7378 |
| 10 | 0.4873 | 0.8679 | 0.5991 | 0.7088 | 0.8596 |
| 20 | 0.5172 | 0.9347 | 0.9597 | 0.8885 | 0.9664 |
| 40 | 0.5528 | 0.9724 | 0.9767 | 0.9701 | 0.9797 |
| 60 | 0.5690 | 0.9816 | 0.9819 | 0.9755 | 0.9872 |
| 80 | 0.5831 | 0.9899 | 0.9823 | 0.9822 | 0.9942 |
| 100 | 0.6079 | 0.9866 | 0.9882 | 0.9919 | 0.9965 |
